# Ultralow Noise Orthogonal Fluxgates Enabling Weak Magnetic Field and Biomolecular Detection

**DOI:** 10.1007/s40820-026-02317-2

**Published:** 2026-08-03

**Authors:** Xiaofeng Pu, Zhijun Sheng, Zhoulu Yu, Huaidong Li, Xintao Wei, Tao Yang, Qingfang Liu, Guozhi Chai, Xiaolei Liang, Daqiang Gao

**Affiliations:** 1 Key Laboratory of Magnetism and Magnetic Functional Materials (Lanzhou University), Ministry of Education, Lanzhou, 730000 People’s Republic of China; 2 Department of Obstetrics and Gynecology, Key Laboratory for Gynecologic Oncology Gansu Province, The First Hospital of Lanzhou University, Lanzhou, 730000 People’s Republic of China

**Keywords:** Orthogonal fluxgate, Composite cores, Amorphous-nanocrystalline, Biomagnetic sensing, Alpha-fetoprotein

## Abstract

Noninvasive detection of ultra-weak biomagnetic signals is crucial for modern biosensing, but conventional magnetic sensors are fundamentally limited by intrinsic noise. Orthogonal fluxgates (OFGs) enable high signal-to-noise-ratio detection of weak magnetic fields, yet their performance is still constrained by intrinsic 1/f noise and Barkhausen-related magnetic fluctuations. This study reports a low-noise orthogonal fluxgate in which a newly designed magnetic core and circuit mitigate existing noise limitations. The CoP/Ag composite core, featuring an amorphous-nanocrystalline dual phase, is associated with reduced low-frequency magnetic loss and improved noise performance. When integrated with a closed-loop feedback, the sensor achieves a noise floor of 8 pT/√Hz at 1 Hz. The sensor enables reliable detection of ultralow-concentration magnetic-bead signals. Alpha-fetoprotein (AFP) was used as a model biomarker in an immunomagnetic bead assay, yielding a linear response from 50 fg mL^−1^ to 100 ng mL^−1^ and a detection limit of 50 fg mL^−1^, which compares favorably with representative reported AFP magnetic biosensors. OFGs demonstrate strong prospects in biosensing, geomagnetic measurements, and weak field detection.

**Supplementary Information:**

The online version contains supplementary material available at 10.1007/s40820-026-02317-2.

## Highlights

- A low-noise orthogonal fluxgate was developed using a CoP/Ag composite core with an amorphous-nanocrystalline dual-phase structure.
- The amorphous–nanocrystalline core is associated with reduced magnetic loss and improved domain regularity and, together with closed-loop feedback, enables a noise floor of 8 pT/√Hz at 1 Hz.
- The sensor enables Alpha-fetoprotein (AFP) immunomagnetic detection with a linear range of 50 fg mL^−1^–100 ng mL^−1^ and a detection limit of 50 fg mL^−1^, demonstrating competitive sensitivity for AFP immunomagnetic detection.

**Supplementary Information:**

The online version contains supplementary material available at 10.1007/s40820-026-02317-2.

## Graphical abstract

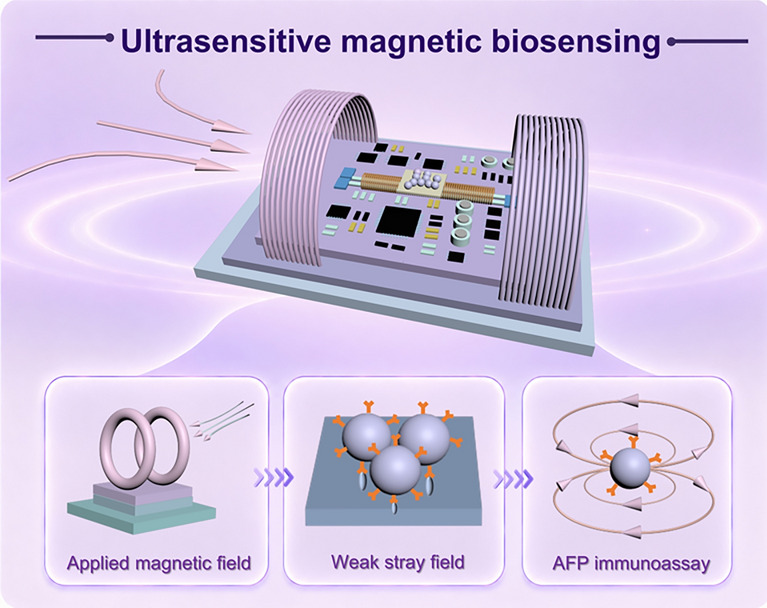

## Introduction

Weak magnetic field detection is of profound significance across a wide spectrum of fields, from fundamental scientific research to applied technology [1]. In the biomedical sector, non-invasive and contactless measurement of extremely weak biomagnetic signals may support the development of future early disease screening and point-of-care diagnostic technologies [2, 3]. Central to this approach is the use of superparamagnetic nanoparticles to label disease-specific biomolecules, such as cancer biomarkers, inflammatory mediators, and pathogenic antigens. This method effectively transduces biological information into a quantifiable magnetic signal, forming the basis for magnetic immunoassays that are both highly sensitive and non-destructive [4–6].

To implement such assays, a variety of magnetic sensing platforms have been explored, each offering distinct advantages in specific contexts. For instance, Hall sensors [7], superconducting quantum interference devices (SQUIDs) [8–10], tunneling magnetoresistance (TMR) sensors [11], and giant magnetoimpedance (GMI) sensors have all been utilized in biodetection applications [12, 13]. Noteworthy examples include the work of Mu et al., who employed a TMR sensor to detect ricin labeled with magnetic nanoparticles across a concentration range of 1 ng mL^−1^ to 200 μg mL^−1^ [14]. Similarly, Sayad et al. used a GMI sensor to quantify the stroke biomarker plasma glial fibrillary acidic protein in patient serum via magnetic bead labeling, achieving a detection limit of 1 ng mL^−1^ [15]. Despite significant advances in magnetic detection technologies, several major limitations persist. While SQUID sensors deliver exceptional sensitivity, their need for cryogenic cooling restricts portability and practical deployment. On the other hand, TMR sensors offer advantages in miniaturization and high-sensitivity magnetic readout; however, under certain device configurations and biasing conditions, their nonlinear transfer characteristics and limited linear operating range may complicate quantitative weak-field detection, particularly for DC or quasi-static magnetic-field measurements [16, 17]. GMR sensors are also suitable for miniaturized magnetic sensing, but their low-frequency performance is often affected by excess 1/*f* noise and by magnetic and electronic fluctuations [18, 19]. Therefore, room-temperature magnetic sensors combining high linearity, suppressed low-frequency noise, and compatibility with biomolecular detection remain highly desirable.

**Fig. 1:**
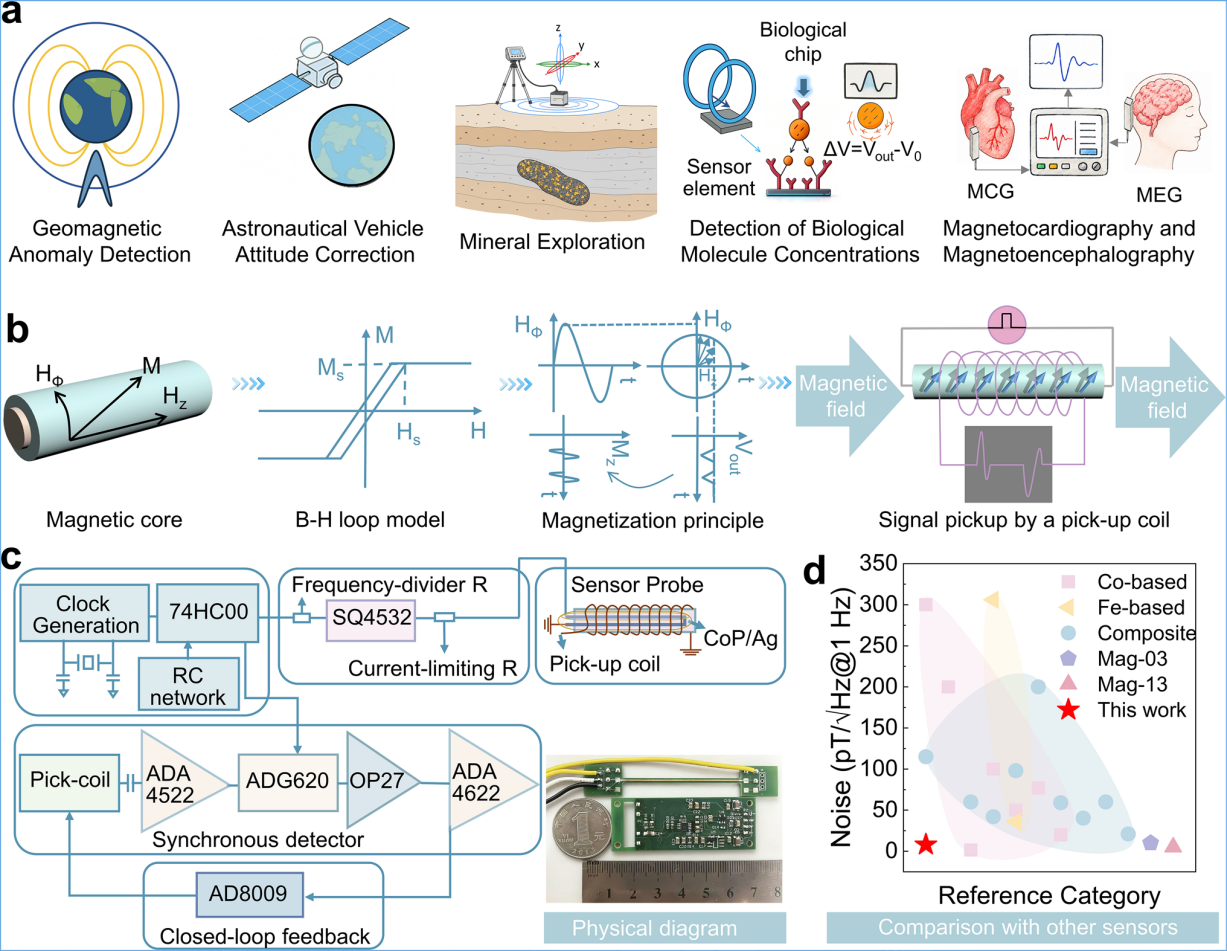
Overview of the OFG: from working principle and circuit realization to microstructural architecture of the CoP/Ag composite core. **a** Application scenarios. **b** Working principle and physical diagram of the OFG. **c** Integrated OFG circuit module and assembled sensor prototype. **d** Comparison of noise performance among sensors with different core types and commercial sensors (Mag-03 and Mag-13, Bartington, UK)

Born from the need to detect weak magnetic fields, Orthogonal Fluxgates (OFGs) are, in principle, an ideal compromise, characterized by high sensitivity, superior noise performance, and outstanding linearity [20–23]. These properties have enabled their use in areas such as geomagnetic anomaly detection [24, 25], spacecraft attitude control [26], mineral exploration [27], and biomolecular detection [28], as well as in high-precision measurements of extremely weak biomagnetic signals such as magnetocardiography and magnetoencephalography [29, 30] (Fig. 1a). Nevertheless, their application in biomagnetic sensing, particularly for molecular recognition and quantitative assays using magnetic bead (MB) labels, has remained largely underdeveloped. Realizing this potential will require coordinated improvements at both the material and device levels, opening pathways to highly sensitive, room temperature biosensing. Notably, a fundamental challenge in this context is the reliable detection of ultra-low target concentrations, where magnetic beads are sparse and signals fall to pT levels or below, often obscured by intrinsic sensor noise and environmental interference. The ultimate resolution of such sensors is constrained by low-frequency magnetic noise originating largely from the core material itself, including an unavoidable 1/*f*-type component. This noise is mainly associated with Barkhausen jumps caused by domain-wall pinning, irreversible magnetization in unsaturated regions, and eddy-current losses [31–34]. Consequently, the core’s magnetic properties constitute a primary bottleneck in overall noise performance, making further improvements in detection sensitivity dependent on the development of ultralow-noise magnetic cores, together with optimized excitation and readout circuitry.

Conventional OFG core materials are generally divided into two categories. Homogeneous amorphous wires offer low coercivity and high permeability, which partially suppress magnetization noise. However, their shell-core domain structure limits central saturation and increases low-frequency noise [35]. Alternatively, FeNi-plated composite cores provide high conductivity [36], which reduces unsaturated regions and skin effects, but their coarse crystalline texture leads to large domain spacing and limited permeability, impairing soft magnetic performance [37]. To overcome these limitations, we designed a novel amorphous-composite magnetic core consisting of a CoP-based amorphous-nanocrystalline coating on a silver substrate. This configuration effectively combines the advantages of both conventional core types. The core is fabricated through a simple and low-cost process, which significantly enhances its potential for commercialization. Our approach follows a three stage strategy: 1) an amorphous CoP layer prepared by electrochemical deposition can reduce the density of large-scale grain boundaries, thereby potentially weakening domain-wall pinning and Barkhausen jumps; 2) controlled DC annealing introduces nanocrystals (10–20 nm) within the amorphous matrix to enhance structural order while retaining high permeability; and 3) precise control of magnetic layer thickness and domain size, combined with optimized coil and circuit design, contributes to noise reduction at both material and system levels, thereby mitigating the core-related noise limitation described above.

Guided by these principles and based on the operating mechanism of the OFG (Fig. 1b), we systematically optimized electrodeposition current density, annealing current, and CoP layer thickness, and implemented a closed-loop feedback signal conditioning circuit to achieve coordinated microstructure modulation (Fig. 1c), device integration, and noise characterization. An optimized noise floor of 8 pT/√Hz at 1 Hz and a sensitivity of 153.8 kV T^−1^ were achieved, showing highly competitive noise performance among reported composite-core-based OFGs (Fig. 1d). To further clarify the performance characteristics of this device in weak-field sensing, Table S1 summarizes the equivalent magnetic noise and typical working conditions of recent representative magnetic sensing technologies. The CoP/Ag-based OFG achieves a system-level input-referred equivalent magnetic noise of 8 pT/√Hz at 1 Hz under room-temperature closed-loop operation, while retaining the advantages of miniaturization and room-temperature operation, demonstrating its overall potential for low-frequency weak field sensing and biomagnetic detection. Building on this performance, we systematically evaluated the sensor response to superparamagnetic magnetic beads with diameters ranging from 100 nm to 1 µm. Under highly diluted conditions, the OFG produced distinguishable magnetic responses above the noise floor, demonstrating its capability for ultralow-concentration magnetic-bead detection. To assess its biosensing performance, alpha-fetoprotein (AFP) was selected as a biomarker and detected via an immunomagnetic bead assay. The sensor exhibits a well-defined linear response to AFP concentrations from 50 fg mL^−1^ to 100 ng mL^−1^ and achieves a detection limit of 50 fg mL^−1^, demonstrating competitive sensitivity within the present OFG-based AFP immunomagnetic detection configuration and providing a viable route toward practical low-frequency weak magnetic sensing.

## Experimental Section

### Preparation of Sensing Elements

The composite structured core was fabricated by electrochemical deposition. A highly conductive silver core with a diameter of 100 μm was selected as the inner core, onto which an amorphous CoP coating was deposited. The electrolyte was based on a phosphate hypophosphite system containing Co^2+^ and hypophosphite ions, with a typical composition of 0.5 mol L^−1^ CoSO₄·6H₂O, 0.2 mol L^−1^ NaH₂PO₂·6H₂O, 0.2 mol L^−1^ Na₃C₆H₅O₇·2H₂O, 0.15 mol L^−1^ C_4_H_4_Na_2_O_6_·2H_2_O, and 0.5 mol L^−1^ (NH4)_2_SO_4_. The Co to P ratio in the deposited layer was controlled by adjusting the current density, which was set to 0.8, 1.0, and 1.2 A dm^−2^ to obtain CoP coatings with different ratios. Under the optimal condition of 1.0 A dm^−2^, the electrodeposited layer exhibited a composition of Co_86_P_14_ (Detailed data are shown in Tables S2-S4), displaying excellent soft magnetic properties.

During electrodeposition, the U-shaped silver core was fixed on the electrode holder and immersed in the electrolyte. To ensure controllable coating thickness, the final thickness of the magnetic layer was adjusted by regulating the deposition time (e.g., 7500 s yielded a thickness of approximately 16 μm). After deposition, the samples were thoroughly rinsed with deionized water and dried.

To optimize the magnetic performance, selected samples were further subjected to current annealing in a DC annealing setup, with annealing currents of 1.6, 1.8, 1.9, and 2.1 A applied for 15 min. Therefore, annealing is described by the applied DC current and duration, with relevant Co-P structural evolution temperatures provided in the Supporting Information for reference. The axial DC annealing current flowing through the CoP/Ag core generates a self-induced circumferential magnetic field according to Ampère’s law; therefore, the annealing process involves both Joule heating and the circumferential field generated by the current itself.

### Design and Manufacture of Sensor

As shown in Fig. 1b, we designed and fabricated an OFG based on a CoP/Ag composite core. To reduce noise and enhance mechanical stability, the magnetic core was shaped into a U-shaped and fixed onto a custom U-shaped printed circuit board (PCB) frame by high-precision soldering. This structural design not only ensured the symmetry and reproducibility of the core, but also effectively suppressed noise sources arising from parasitic and non-uniform stresses.

A pickup coil was wound around the magnetic core using enameled copper wire with a total of 1600 turns, tightly wound to maximize space utilization and magnetic coupling efficiency. To further reduce thermal noise, low resistivity copper was employed, and the winding tension was precisely controlled to minimize mechanical stress during fabrication.

The probe was encapsulated in a glass capillary, which provided insulation and mechanical fixation while protecting the sensor performance from ambient humidity and external disturbances. In addition, the low dielectric constant of the glass reduced capacitive coupling between the coil and the external environment, thereby suppressing low-frequency noise. The PCB frame was further designed with reserved input and output ports for the excitation current and pickup-coil signal, enabling direct integration with external driving circuitry.

### Structural, Magnetic, and Sensing Characterization

Focused ion beam (FIB) testing was performed using a focused ion beam system (Thermo Scientific Helios 5 CX), while transmission electron microscopy (TEM) characterization was performed using a Talos F200S microscope equipped with a Super-X EDS detector. The elemental composition of the CoP layer was determined by TEM-EDS area analysis on selected coating regions to minimize the contribution from Ag. Scanning electron microscope (SEM) images were acquired using a Thermo Scientific Apreo’S scanning electron microscope. The static hysteresis loops were measured using a BH-Loop analyzer (BHV-30S), with the external magnetic field applied along the axial direction of the CoP/Ag composite wire. The schematic diagram of the dynamic circumferential hysteresis-loop measurement for the magnetic core is shown in Fig. S5. The magnetic domain structure of the magnetic core was characterized using magnetic force microscopy (MFM, Bruker Dimension Icon) equipped with a MEAP probe. All MFM images were acquired at room temperature under zero external magnetic field, and therefore represent the zero-field remanent domain states after the corresponding deposition or annealing treatments. Considering the curved microwire geometry of the CoP/Ag core and the local nature of the domain contrast, MFM was used to obtain surface stray-field contrast with high spatial resolution. The measurements were performed in tapping mode (The brightness and color contrast originate from variations in the MFM phase signal induced by the local stray magnetic field on the sample surface, reflecting the magnetic domain distribution characteristics of different samples). X-ray photoelectron spectroscopy (XPS) was carried out using a Kratos AXIS Ultra DLD instrument, and Fourier transform infrared (FTIR) spectra were recorded with a Nicolet iS50 spectrometer. To evaluate the sensor’s noise performance, a measurement setup based on a Brüel & Kjær data acquisition system was established. The sensor probe was connected to a custom designed circuit board integrating the excitation circuitry, low-noise amplification, and DC signal conversion modules, enabling excitation, signal amplification, and DC output processing within a single board. The resulting DC voltage signal was fed directly into the high-precision Brüel & Kjær data acquisition system for real time acquisition and storage. The onboard analysis modules were then used to process the voltage signal, and the equivalent magnetic noise was calculated from the power spectral density. All measurements were conducted inside a five-layer magnetic shielding cylinder to effectively suppress environmental magnetic interference, ensuring the accuracy and reproducibility of the noise characterization.

## Results and Discussion

### Operating Principle of Sensors and Structural Control of Amorphous-Nanocrystalline Magnetic Cores

Figure 1b illustrates the structure and operating principle of the OFG probe, which employs circumferential AC excitation and axial magnetic-field pick-up. The excitation coil generates an alternating circumferential magnetic field within the CoP/Ag composite core, whereas the measured external field is a DC/quasi-static axial magnetic field applied along the core direction. In the calibration measurements, the external field was set within the geomagnetic-field range, representing a typical weak-field condition for OFG operation. This axial field modulates the magnetization process of the core and generates a pick-up voltage proportional to the external magnetic field, enabling linear weak-field detection. The pick-up voltage spectrum was synchronously demodulated to yield a DC signal, facilitated by the integrated drive amplification demodulation circuitry, with the assembled device shown in the Fig. 1c. The device is a miniaturized OFG, highlighting its potential for deployment in miniaturized electronic systems and space-constrained applications. Figure S2a-d characterize the macroscopic morphology and nanoscale phase evolution of the CoP/Ag composite core. Here, the optimally annealed sample refers to the CoP/Ag core prepared at an electrodeposition current density of 1.0 A dm^−2^ for 7500 s and subsequently treated by current-assisted annealing at 1.8 A for 15 min. The scanning electron microscope (SEM) cross-sectional and surface images of the composite core shown in Fig. S2a, b reveal that the CoP coating forms a continuous and dense layer over the silver core substrate, without visible cracks or delamination. The weak layered contrast in the cross-section mainly reflects radial deposition contrast induced by growth variation during electrodeposition, rather than crystalline lamellae. The nanoscale structural state of the CoP layer was further determined by high-resolution TEM (HRTEM) and selected area electron diffraction (SAED). These observations indicate excellent coverage and interfacial adhesion from the electrodeposition process, which are critical for achieving magnetic flux closure and long-term device stability. In the unannealed CoP layer (Fig. S2c), the HRTEM image acquired from the CoP coating region shows uniform contrast without long-range lattice fringes, and the corresponding SAED pattern displays diffuse halos, indicating an amorphous or short-range ordered structure. After current-assisted annealing (Fig. S2d), local nanocrystalline clusters emerge within the CoP coating, as marked by blue dashed contours, and the coating-region SAED pattern exhibits clearer diffraction rings. These results indicate a structural evolution of the CoP layer from an amorphous state toward an amorphous–nanocrystalline dual-phase structure [38, 39]. XRD patterns in Fig. S3 remain dominated by a broad amorphous background before and after annealing, indicating that the CoP coating does not undergo overall crystallization. In contrast, the locally clearer lattice fringes in HRTEM and the more distinct diffraction rings in SAED after annealing reveal enhanced local ordering and partial nanocrystallization. The surface chemical states and oxygen-content variation of the CoP coating before and after annealing were further examined by XPS, as discussed in the Supporting Information. This demonstrates that the carefully optimized annealing conditions induce partial nano-crystallization without disrupting the overall amorphous matrix, producing a microstructure that enhances magnetic performance while preserving low coercivity and minimal noise.

**Fig. 2:**
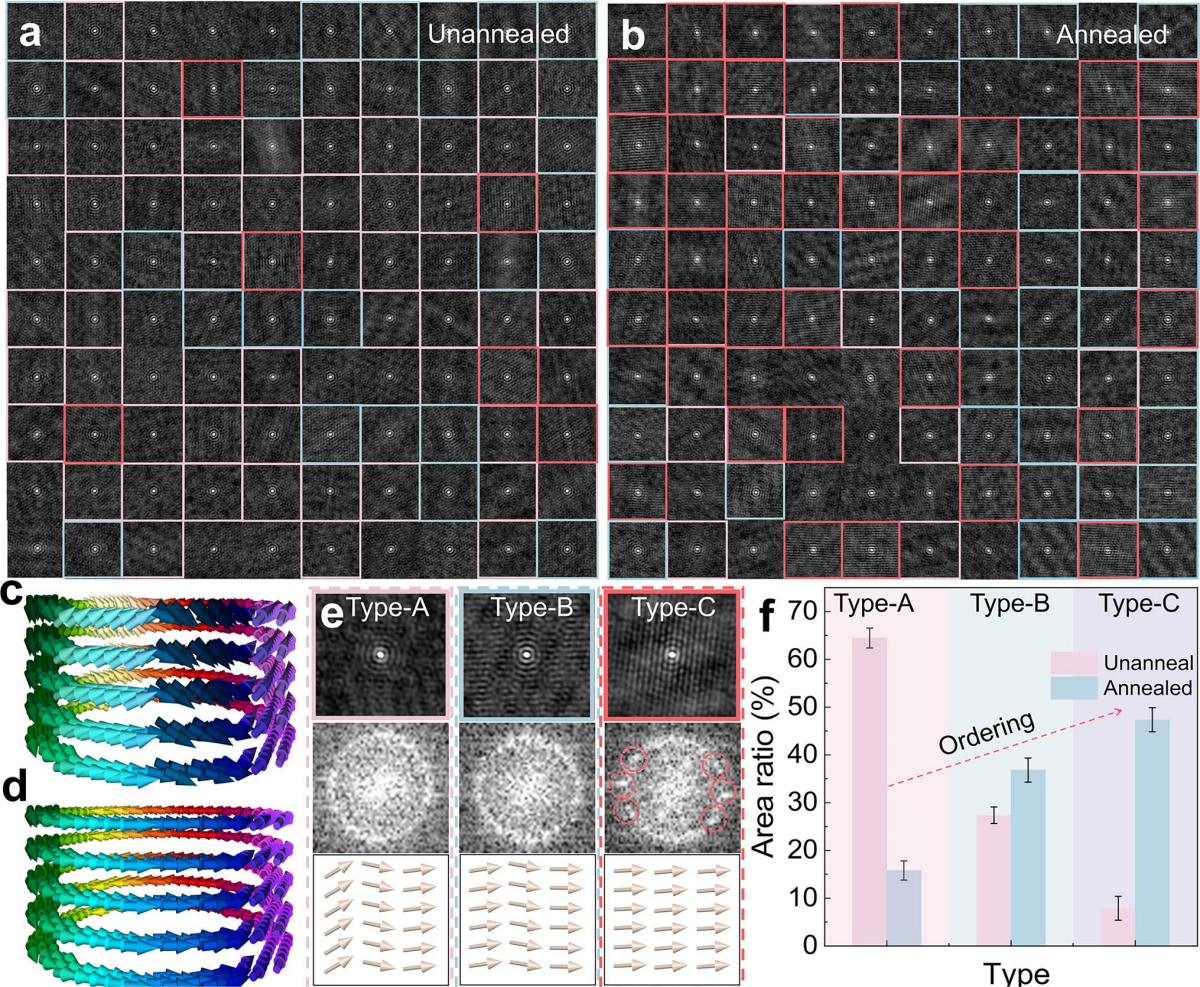
Microstructural ordering transition and magnetic moment alignment induced by annealing. **a** Autocorrelation function before annealing. **b** Autocorrelation function after annealing. **c** Magnetization state in micromagnetic simulations before introducing the dual-phase structure. **d** Magnetization state in micromagnetic simulations after introducing the dual-phase structure. **e** FFT images of Types A-C and their corresponding magnetic moment states. **f** Total area fractions of Types A, B, and C before and after annealing

Figure 2 systematically illustrates the microstructural evolution of the CoP/Ag composite core from a disordered to an ordered state during current assisted annealing (The samples analyzed in Fig. 2 were deposited at 1.0 A dm^−2^ for 7500 s; the annealed sample was further treated at 1.8 A for 15 min). Figure 2a, b presents high-resolution TEM images of the CoP/Ag composite layer before and after current assisted annealing, accompanied by autocorrelation function (ACF) analyses. Using pixel by pixel Fourier transform and local autocorrelation analysis, three distinct structural types are identified: Type-A (pink box), corresponds to dispersed disordered regions with blurred lattice fringes and negligible periodicity; Type-B (blue box), showing weak periodic features indicative of short-range ordered nanocrystal clusters; Type-C (red box), displaying well-defined lattice orientation and periodic modulation characteristic of long-range ordered grain clusters. In the as-deposited sample (Fig. 2a), the structure is dominated by Type-A and Type-B regions, with few long-range ordered areas. After annealing (Fig. 2b), the ratio of Type-C increases significantly (7.9% to 47.4%), indicating that annealing promotes significant structural rearrangement and ordering. To further elucidate how lattice ordering influences magnetization behavior, micromagnetic simulations were performed to visualize the magnetic moment state before and after introducing the dual-phase structure (In the simulated results, the arrows indicate the local normalized magnetization directions, while the colors represent the spatial variation in magnetic-moment orientation). As shown in Fig. 2c, prior to the incorporation of the dual-phase architecture, the magnetization exhibits locally nonuniform rotation characterized by angular mismatch between adjacent regions. Further details of the simulation, including the magnetic-moment representation and its consistency with Maxwell’s equations, are provided in the Supporting Information. Scaled models were used only for qualitative comparison under identical conditions to reveal relative changes in magnetization-rotation uniformity. The magnetic moments respond to excitation in a segmented and partially decoupled manner. In contrast, after introducing the dual-phase structure and enhanced lattice ordering (Fig. 2d), the magnetization evolves into a more continuous and coordinated rotational state. The angular variation between neighboring regions becomes smoother. Since domain-wall motion and local irreversible magnetization may still exist in the composite core, this coordinated response should not be regarded as ideal coherent rotation. Rather, improved structural ordering weakens local pinning and nonuniform rotation, thereby promoting a low-loss quasi-coherent rotational tendency. It should be noted that annealing does not affect the magnetic response only through microstructural ordering. Local nanocrystalline clusters and clearer periodic features may also change the local magnetocrystalline anisotropy and the energy barrier for magnetization rotation. In amorphous–nanocrystalline soft magnetic systems, fine and dispersed nanocrystals can average randomly oriented local anisotropy through exchange coupling. This reduces the effective anisotropy and promotes a more uniform magnetic response [40]. However, excessive annealing may cause grain coarsening or local anisotropy mismatch, leading to stronger magnetization inhomogeneity and pinning [40–42]. Therefore, the improved magnetic response after optimized annealing is attributed to the combined effects of structural ordering, local anisotropy modulation, and stress relaxation.

The schematic in Fig. 2e further depicts the magnetic moment state associated with each structural type: the magnetic moment state evolves from random, uncorrelated orientations in Type-A to short-range correlated alignment in Type-B, and in Type-C regions, the clearer lattice orientation and periodic modulation are expected to promote a more cooperative alignment of magnetic moments [43, 44]. This progressive ordering is expected to promote a more coordinated magnetic response under excitation. If the magnetization response becomes more coordinated, the associated irreversible magnetic processes and magnetic loss are expected to decrease. In OFGs, an important contribution to low-frequency magnetic noise is associated with magnetization fluctuations, including nonuniform rotation, irreversible magnetization processes, and magnetic loss. The corresponding spectral density can be approximately described by Eq. (1) [45, 46]:

1 $${b}^{2}\left(f\right)=\frac{{\mu}_{0}}{{sin}^{2}{\theta}_{M}}\frac{4{K}_{B}T}{2\pi fV}\frac{{{H}^{2}}_{int}}{{{M}^{2}}_{s}}\chi {\prime}{\prime}(f)$$

where ${\theta}_{M}$ represents the angle between the magnetization vector and the core axis, while ${\chi }^{{\prime}{\prime}}\left(f\right)$ corresponds to the loss related imaginary component of the dynamic permeability. According to Eq. (1), the magnetic noise is jointly affected by the orientation factor ${1/sin}^{2}{\theta}_{M}$, internal field fluctuation ${H}_{int}$, and loss-related term ${\chi }^{{\prime}{\prime}}\left(f\right)$. When the magnetization approaches transverse saturation (${\theta}_{M}=90^\circ$), ${sin}^{2}{\theta}_{M}$ is maximized, thereby minimizing the influence of the orientation factor on ${b}^{2}\left(f\right)$, provided that the other parameters remain unchanged. More importantly, reduced irreversible magnetic loss and local magnetic inhomogeneity can lower ${H}_{int}$ fluctuations and the effective ${\chi }^{{\prime}{\prime}}\left(f\right)$, thereby reducing noise.

Thermal annealing promotes a more ordered magnetic configuration, which facilitates a more synchronized magnetization rotation [47]. This structural evolution may reduce local fluctuations in the internal field ${H}_{int}$ and lower the effective magnetic loss contribution ${\chi }^{{\prime}{\prime}}\left(f\right)$, thereby contributing to the reduction of noise. This interpretation is consistent with the reduced circumferential dynamic hysteresis loop area after annealing, from 4.34 × 10^−6^ to 2.52 × 10^−6^ Wb mA^−1^, as well as with the lower equivalent magnetic noise at 1 Hz (Fig. 4a). Together, these results suggest that microstructural ordering is closely associated with the improved noise performance. A quantitative comparison linking the Type-C ordered-region fraction, circumferential dynamic magnetic loss, and 1 Hz equivalent magnetic noise is provided in Table S9 and further discussed in the Supporting Information.

### Analysis of Soft Magnetic Properties of Magnetic Cores Under Different Deposition Conditions

**Fig. 3:**
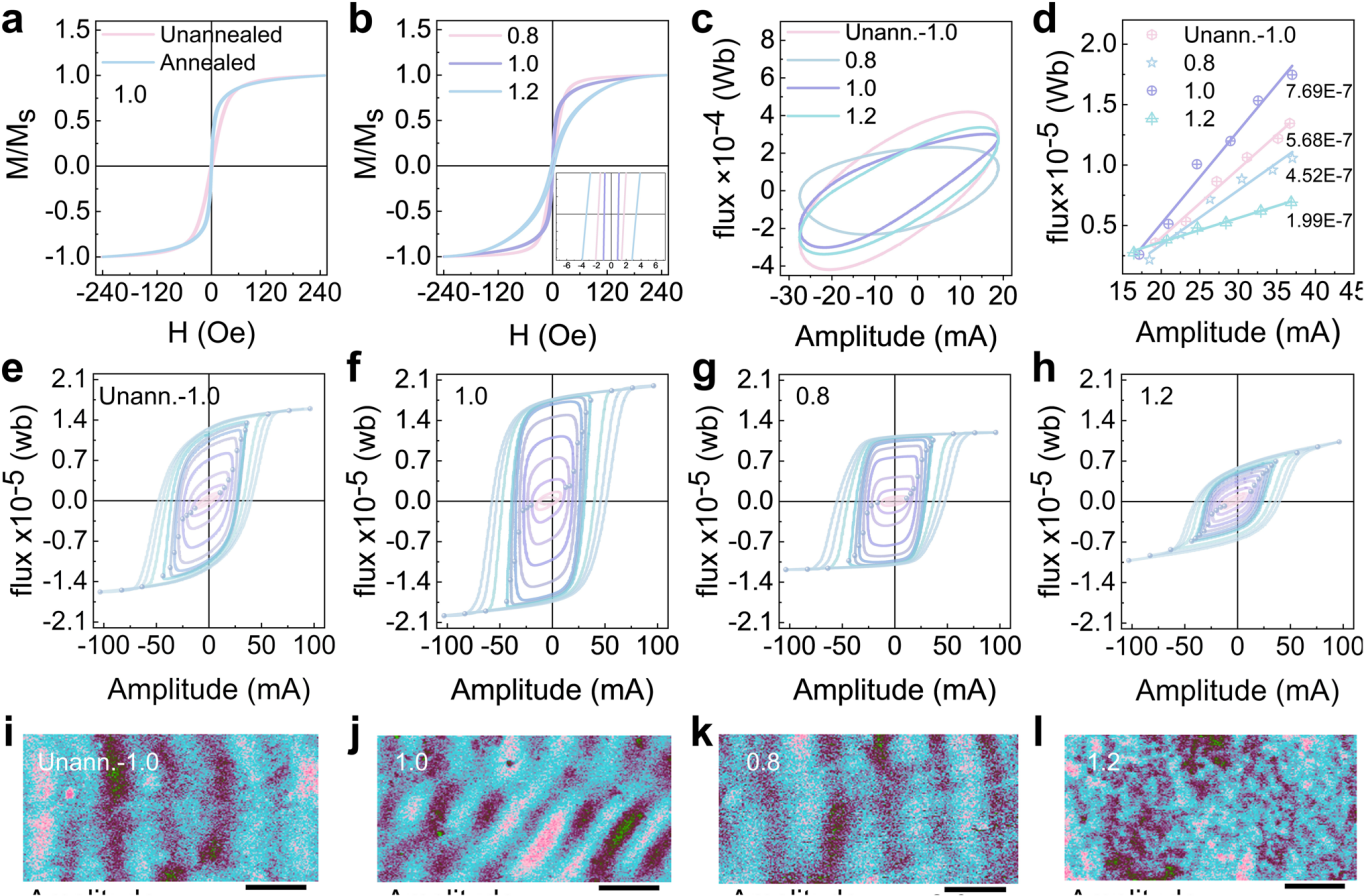
Characterization of soft magnetic properties of CoP/Ag composite core. **a** Comparison of hysteresis loops before and after current-assisted annealing at 1.0 A dm^−2^. **b** Magnetic hysteresis loops under different deposition current densities. **c** Circumferential dynamic hysteresis loops under different deposition current densities. **d** Comparison of initial magnetization rates of circumferential dynamic hysteresis loops for different current densities. **e–h** Circumferential dynamic hysteresis loops of different deposition current densities under 10 kHz, 0 mA bias, amplitude varying excitation conditions; the annealed 1.0 A dm^−2^ sample was treated at 1.8 A for 15 min. **i-l** Magnetic domain conditions under different deposition current densities

Unless otherwise specified, the annealed 1.0 A dm^−2^ sample discussed in this section refers to the CoP/Ag core annealed at 1.8 A for 15 min. The hysteresis-loop shape, coercivity, initial susceptibility, and dynamic loop area reflect the combined effects of microstructure, local magnetic anisotropy, residual stress, and defect pinning. During electrodeposition, the CoP coating grows on the Ag core and cannot expand or shrink completely freely during deposition and annealing, which may introduce residual stress. This stress can generate additional effective anisotropy through magnetoelastic coupling and enhance domain-wall pinning. Current-assisted annealing may partially release this stress, reduce irreversible magnetic loss, and promote more stable magnetization rotation. Therefore, lower coercivity, higher initial susceptibility, and a smaller dynamic loop area are generally favorable for reducing low-frequency magnetic noise in OFG cores. Figure 3a compares the hysteresis loops of the 1.0 A dm^−2^ sample before and after annealing. The annealed sample exhibits a steeper loop and reduced coercivity, indicating that magnetization reversal occurs more readily under low applied fields, a result of decreased internal stress and structural defects, which enhance domain wall mobility [48]. Figure 3b presents hysteresis loops of samples deposited at different current densities. The sample deposited at 1.0 A dm^−2^ exhibits the best soft magnetic properties, with a narrow loop that saturates rapidly, reflecting uniform magnetic reversal. In contrast, the 0.8 A dm^−2^ sample shows higher coercivity, suggesting defect-induced pinning of domain walls, while the 1.2 A dm^−2^ sample displays incomplete saturation, due to stress and inhomogeneity caused by excessive deposition rate. These results identify 1.0 A dm^−2^ as the optimal deposition condition, balancing low coercivity and high permeability. Figure 3c shows the circumferential dynamic hysteresis loops under different deposition conditions. The largest loop area (4.34E-6 Wb mA^−1^ is observed in the unannealed sample, indicating significant magnetic loss response ${\chi }^{{\prime}{\prime}}\left(f\right)$ and irreversible magnetization caused by internal stress and structural defects [49]. The 0.8 A dm^−2^ sample shows a slightly reduced loop area (3.12E-6 Wb mA^−1^), though domain structure remains nonuniform due to local stress concentration. The smallest loop area (2.52E-6 Wb mA^−1^) is achieved in the annealed 1.0 A dm^−2^ sample, consistent with its uniform amorphous-nanocrystalline dual-phase structure and minimal magnetic loss. At 1.2 A dm^−2^, the loop area increases again (3.00E-6 Wb mA^−1^), implying that high deposition rates introduce stress induced losses.

Furthermore, the circumferential dynamic hysteresis-loop results measured at 10 kHz (Fig. 3d–h) reveal the key factors that govern the performance of OFGs. Figure 3d summarizes the initial susceptibilities (loop slopes) extracted from the curves shown in Fig. 3e–h under different deposition conditions. The sample deposited at 1.0 A dm^−2^ exhibits the highest initial susceptibility, about 7.69 × 10⁻⁷ Wb mA^−1^, which is substantially larger than those of the 0.8 and 1.2 A dm^−2^ samples. The initial susceptibility directly reflects the permeability of the sample under weak excitation (10 kHz), generally expressed as ${\mu}_{init}={\left.\frac{dM}{dH}\right|}_{H\to 0}$, where a larger ${\mu}_{init}$ indicates that magnetic moments can deflect more easily and more uniformly under very small excitation. The annealed 1.0 A dm^−2^ sample shows the highest ${\mu}_{init}$, implying that under identical driving conditions it can produce a larger output signal while reducing irreversible magnetization contributions. This behavior is consistent with the improved signal-to-noise ratio and the lower 1 Hz noise level.

Furthermore, the influence of the circumferential dynamic hysteresis loop shape on noise was analyzed [21, 50]. The annealed 1.0 A dm^−2^ sample (Fig. 3e, f) exhibits a more rectangular and symmetric circumferential dynamic hysteresis loop with a larger dynamic flux amplitude, indicating an enhanced effective magnetic response under circumferential excitation [51]. In the dynamic loop measurement, the recorded flux amplitude reflects the magnetic flux coupled to the pick-up coil, which is jointly affected by the intrinsic magnetization of the magnetic layer, the effective permeability of the core, the magnetically active volume participating in the dynamic response, and the flux-coupling efficiency. Current-assisted annealing promotes structural ordering, partially relieves residual stress, and reduces local pinning inhomogeneity, thereby improving the effective permeability and magnetic flux coupling of the CoP/Ag core. As a result, a larger dynamic flux amplitude can be obtained under the same circumferential excitation. Although the finite loop opening indicates that irreversible magnetization processes remain, the reduced loop area suggests that domain-wall-related loss and local pinning are alleviated after annealing. As a result, magnetic moments can rotate more continuously and uniformly under weak excitation, while the results are consistent with reduced pinning-related irreversible magnetization processes. The smaller loop area therefore reflects the combined contribution of enhanced coordinated moment rotation and reduced domain-wall pinning, consistent with the observed reduction in low-frequency noise. The 0.8 A dm^−2^ sample (Fig. 3g) exhibits a broader loop with higher coercivity, suggesting strong domain wall pinning due to defects and nonuniform crystallization, which increases hysteretic loss and noise. For the 1.2 A dm^−2^ sample (Fig. 3h), the higher deposition current density accelerates CoP layer growth and introduces more pronounced compositional deviation, residual stress, and structural inhomogeneity, thereby enhancing local magnetoelastic anisotropy and restricting continuous moment rotation. Although subsequent annealing can partially relieve residual stress, it cannot fully eliminate the structural and anisotropy inhomogeneities formed during deposition. Consequently, this sample still exhibits larger dynamic loss and higher noise. The Supporting Information provides circumferential dynamic hysteresis loops under various deposition and annealing conditions (Fig. S6), illustrating how different coating thicknesses and annealing currents influence the magnetic properties. Domain structure observations (Fig. 3i–l) further support these conclusions from the perspective of local magnetic-domain distribution [52]. The unannealed 1.0 A dm^−2^ sample (Fig. 3i) shows irregular, ripple like domains, indicative of pinning by defects and local stresses. After annealing (Fig. 3j), more regular striped domains appear, indicating improved domain regularity and suggesting the formation of a more favorable circumferential anisotropy. By contrast, the 0.8 A dm^−2^ sample (Fig. 3k) exhibits coarse and distorted domains, consistent with high defect density, while the 1.2 A dm^−2^ sample (Fig. 3l) shows blurred and disordered domains, reflecting structural instability. Further quantitative comparison of the surface circumferential domain characteristics, together with the origin of the disordered domains in the 1.2 A dm^−2^ sample, is provided in the Supporting Information. Together, these results indicate that the optimized noise performance of the annealed 1.0 A dm^−2^ sample is associated with higher permeability, improved domain regularity, and reduced dynamic magnetic loss. These factors are expected to facilitate a more uniform magnetization response and thereby contribute to the reduction of low-frequency magnetic noise.

### Signal Conditioning Circuit Fundamentals and Comparative Analysis of Core Noise Performance Under Different Deposition Conditions

**Fig. 4:**
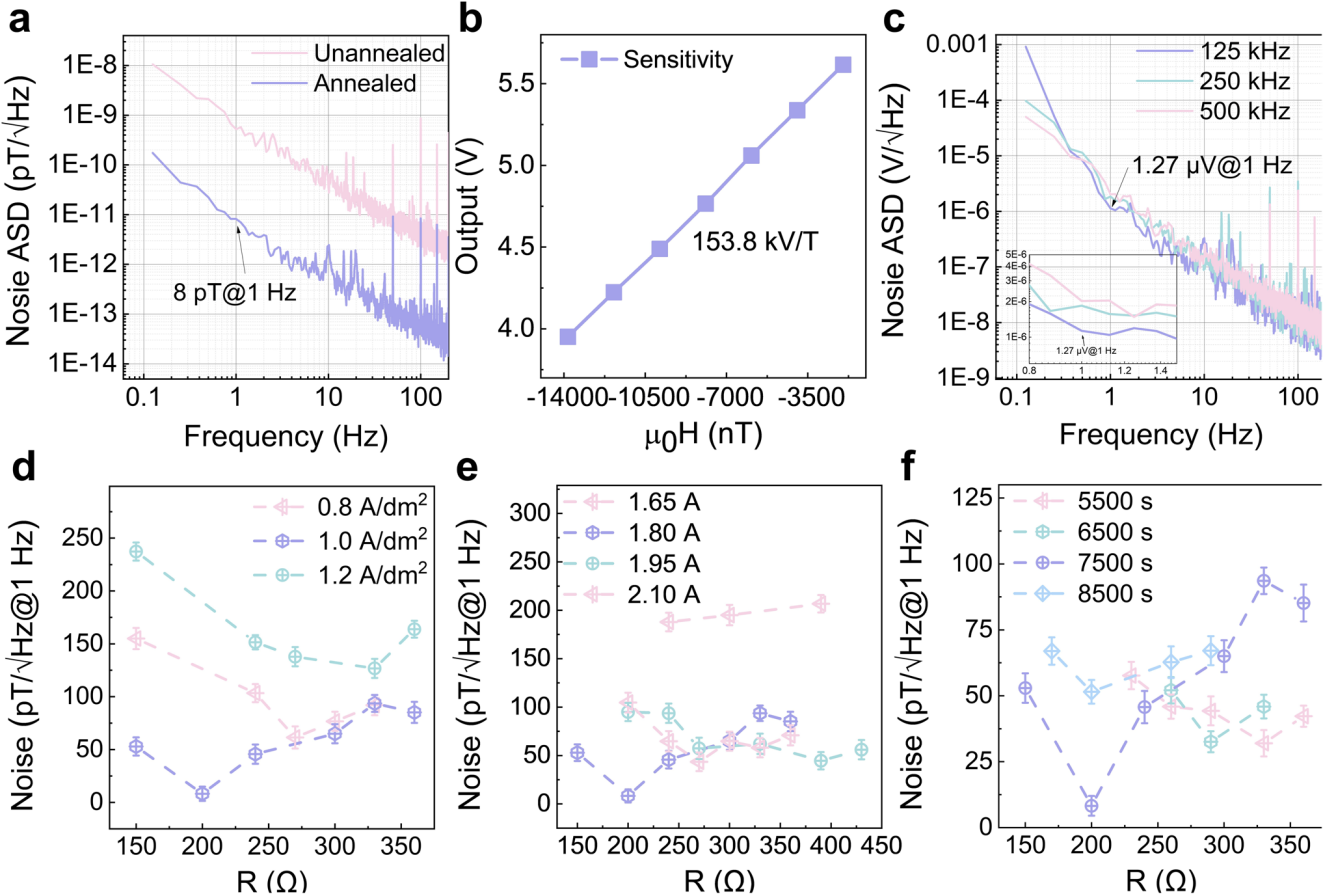
Output characteristics and noise performance of the OFG. **a** Comparison of noise performance between annealed and unannealed magnetic cores. **b** OFG output as a function of the applied magnetic field. **c** Voltage noise spectra of the sensor under different excitation frequencies. **d** 1 Hz noise versus current-limiting resistance R for different deposition current densities, with fixed deposition time of 7500 s and annealing at 1.8 A.** e** 1 Hz noise versus current-limiting resistance R for different annealing currents, using samples deposited at 1.0 A dm^−2^ for 7500 s. **f** 1 Hz noise versus current-limiting resistance R for different deposition times, using samples deposited at 1.0 A dm^−2^ and annealed at 1.8 A

Figure 4 presents the output characteristics, noise spectra, and process-dependent noise optimization of the implemented OFG. Figure 4a compares the equivalent magnetic noise spectra of the unannealed sample and the optimally annealed CoP/Ag core. Unless otherwise specified, the optimized sample refers to the core prepared at an electrodeposition current density of 1.0 A dm^−2^ for 7500 s and subsequently annealed at 1.8 A for 15 min. The noise measurement was performed under closed-loop operation with a 125 kHz square-wave excitation and a 200 Ω series current-limiting resistor. Compared with the unannealed sample, the optimally annealed core exhibits a clear reduction in low-frequency noise, reaching 8 pT/√Hz at 1 Hz. This result is consistent with the reduced dynamic hysteresis loss and improved domain regularity discussed in Fig. 3.

The sensor operates in a closed-loop fluxgate configuration driven by square-wave excitation. A 74HC00 logic gate combined with an RC network generates a stable clock signal, and the measured square-wave waveform is shown in Fig. S8. This signal is amplified by an SQ4532 power MOSFET to provide the excitation current. The ADA4522 precision amplifier and ADG620 synchronous demodulator work in tandem to extract the magnetic-field-dependent signal component while suppressing asynchronous noise and high-frequency interference. Additional amplification by ADA4622 and OP27 expands the dynamic range at low-frequency, while the AD8009 forms part of the feedback loop to compensate for zero drift and enhance voltage linearity. Within this architecture, the sensor output exhibits an excellent linearity with the applied magnetic field (Fig. 4b), achieving a sensitivity of 153.8 kV T^−1^ (For further details on the sensitivity calibration and voltage-to-equivalent-magnetic-noise conversion, please refer to the Supporting Information). The measured -3 dB bandwidth of the OFG sensor was 46 Hz, and the corresponding frequency response data are provided in Table S10. Figure 4c compares the noise spectral density under different excitation frequencies (125, 250, and 500 kHz). All spectra exhibit increased noise density in the low-frequency region, consistent with the typical 1/*f* behavior of fluxgate sensors. Under 125 kHz excitation, the sensor achieves the lowest voltage noise density of 1.27 μV/√Hz at 1 Hz, corresponding to a system-level input-referred equivalent magnetic noise of 8 pT/√Hz (Further discussion of the increased 1 Hz noise under high-frequency excitation is provided in the Supporting Information). The reduction in 1/*f* noise is attributed to the synergistic optimization of the CoP/Ag amorphous-nanocrystalline composite core and the closed-loop readout circuit. Further details on the averaging procedure for the noise spectra in Fig. 4, the voltage-to-equivalent-magnetic-noise conversion, and the reproducibility of repeated noise measurements are provided in the Supporting Information.

This circuit implementation integrates three layers of control, phase stable high-quality excitation, low-noise synchronous demodulation, and closed-loop zeroing to fully translate the intrinsic low-noise potential of the CoP/Ag composite core into device level performance. For further clarification of the circuit contribution in the closed-loop OFG, please refer to the Supporting Information. This performance reflects the combined contribution of material-level and circuit-level optimization. At the material level, nanoscale structural ordering and improved domain regularity are associated with reduced magnetic loss and enhanced permeability. At the circuit level, phase-stable excitation, synchronous demodulation, and closed-loop feedback help suppress electronic noise and improve output stability. Further reductions in noise are expected through differential readout, digital lock-in amplification, and improvements in the front-end electronics.

Since cores prepared under different process conditions exhibit different permeability, magnetization responses, and dynamic losses, a fixed R value does not necessarily correspond to the optimal excitation state for all samples. Therefore, R was scanned for each sample to determine the corresponding optimal excitation current condition and to obtain a more reliable comparison of the 1 Hz noise performance. Figure 4d–f illustrate the noise optimization trends achieved through process parameter control. Electrodeposition current density strongly influences the microstructure of the magnetic layer (Fig. 4d). At 1.0 A dm^−2^, with a Co:P ratio of 86:14, the CoP/Ag core exhibits the most favorable magnetic response, including a uniform domain arrangement and enhanced permeability, thereby achieving the lowest 1 Hz noise level of 8 pT/√Hz. In comparison, the cores prepared at 0.8 and 1.2 A dm^−2^ show higher noise levels of 61 and 126 pT/√Hz, respectively. This noise increase is mainly attributed to non-optimal magnetic responses, such as reduced effective permeability, increased dynamic hysteretic loss, and local domain nonuniformity, rather than a universal increase in coercivity or severe disruption of domain structures in all samples. Annealing effects are particularly significant (Fig. 4e). Moderate annealing (1.8 A) induces nanoscale ordered regions within the amorphous matrix, relieves residual stress, and improves domain regularity. These changes are consistent with the observed reduction in low-frequency noise [53, 54]. Excessive annealing, however, leads to grain coarsening and local anisotropy mismatch, causing noise to increase. Thickness dependence (Fig. 4f) shows an optimal window. A deposition time of 7500 s (16 μm thickness) yields the lowest noise. Thinner layers have insufficient effective permeability, while thicker layers form eddy current paths and increase low-frequency losses.

In Fig. 4d–f, R denotes the series current-limiting resistor in the excitation circuit, which regulates the square-wave excitation current and improves driving stability. It is worth noting that, for each process condition, the series current limiting resistor in the excitation circuit was co-optimized (Each data point was obtained by averaging at least 20 repeated noise spectra measured under identical conditions, and the error bars represent the corresponding measurement variation). Proper resistor selection helps suppress high frequency harmonics in the driving current, reducing parasitic coupling to the pick-up coil, while maintaining a stable excitation waveform. This ensures more uniform magnetization reversal within the core during circumferential magnetization. The circuit optimization synergizes with the improved core microstructure. Under the conditions of 1.0 A dm^−2^ electrodeposition current, 7500 s deposition time, 1.8 A joule annealing current, 200 Ω current limiting resistor, and 125 kHz excitation frequency, the sensor exhibits outstanding noise performance at 1 Hz. As summarized in Fig. 1d, the sensor developed in this work, equipped with the signal-conditioning circuit, exhibits superior noise performance compared with other sensor designs and core types, showing one of the lowest 1 Hz noise levels reported so far among composite-core-based orthogonal fluxgates [21, 37, 55–65]. Representative comparisons with reported fluxgate and related magnetic sensors are provided in Table S11 and further discussed in the Supporting Information. More importantly, this noise level approaches the sensitivity threshold required for detecting biomagnetic signals, such as cardiac, neural, and magnetic bead labeled molecular responses [66, 67]. Furthermore, we demonstrate the sensor’s application in magnetic bead detection, validating its practical feasibility and advantages for biological sensing.

### Response of the OFG to Magnetic Beads of Different Diameters

**Fig. 5:**
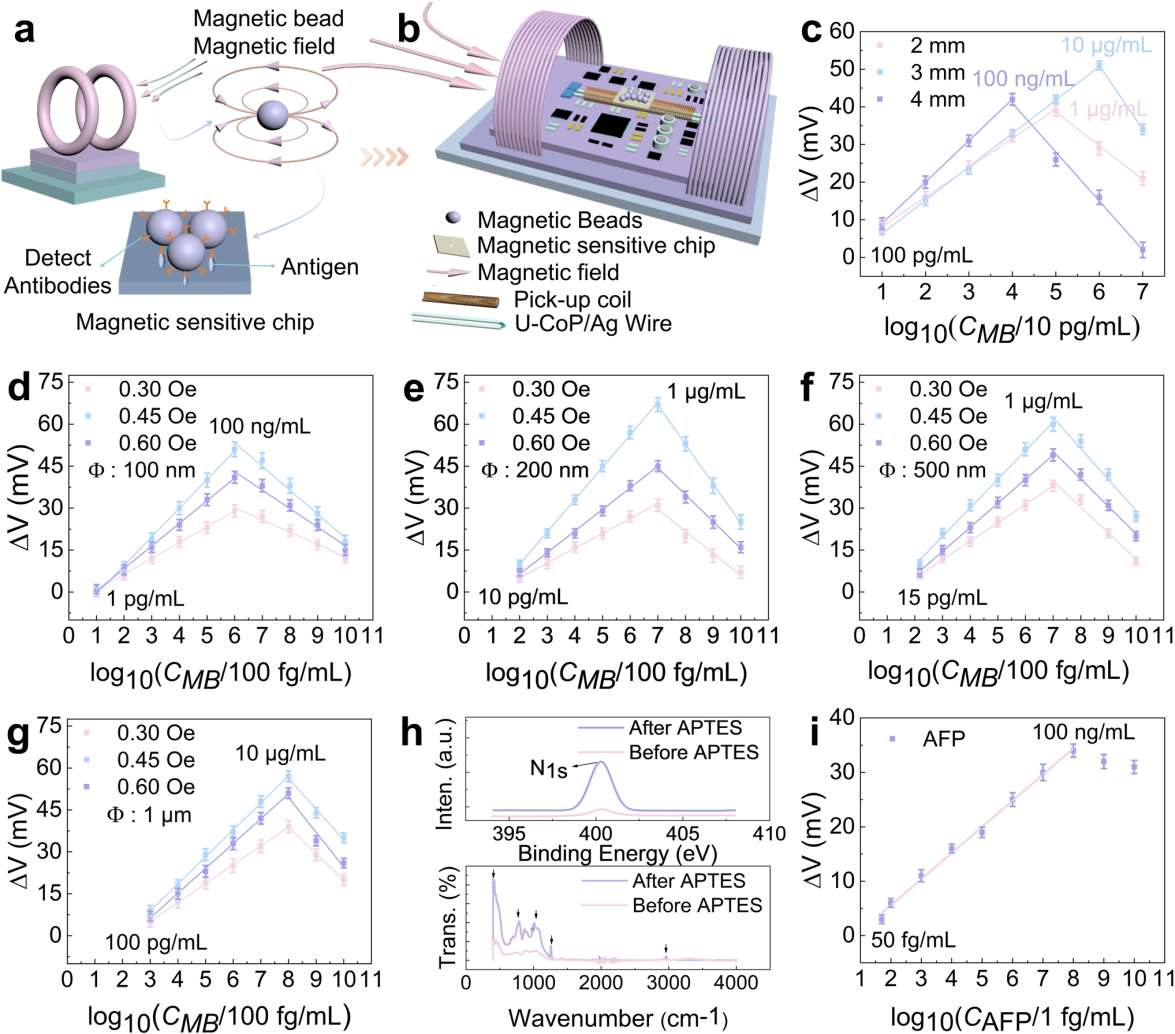
Schematic illustration and measurement results of biological MBs detection. **a** Detection principle: antibody-antigen binding with MBs perturbs the local magnetic field. **b** Device structure: CoP/Ag composite core with pick-up coils on PCB and integrated magnetic sensitive chip for bead-labeled biomolecule detection. **c** Dependence of output voltage on MB concentration for chip apertures of 2 mm, 3 mm, and 4 mm. The variation of output voltage with concentration under different external magnetic field intensities for magnetic beads with different diameters:** d** 100 nm, **e** 200 nm, **f** 500 nm, **g** 1 μm. **h** XPS N 1*s* spectra of the glass surface before and after APTES functionalization (top), and FTIR comparison of glass surfaces with and without 2-h APTES incubation (bottom). **i** Detection results of AFP at different concentrations under an external magnetic field of 0.45 Oe

Figure 5a, b illustrates the detection system and device implemented in this work. The superparamagnetic magnetic beads used in this work are Fe_3_O_4_-based beads consisting mainly of a magnetic Fe_3_O_4_ core and a surface-functionalized coating for biomolecular conjugation. The sensor core consists of CoP/Ag composite core, while the coil and microfluidic channel enable effective coupling with magnetic-bead samples. Under the applied external bias magnetic field, the beads become magnetized, and their local stray fields superimpose on the field distribution sensed by the OFG, thereby modulating the output voltage [68]. To evaluate the ultralow-concentration magnetic-bead detection capability of the OFG, magnetic bead suspensions were serially diluted to a statistically diluted low-bead-number regime. SEM images of the corresponding 200 nm and 1 µm diameter magnetic beads at different concentrations are shown in Figs. S11 and S12. The test solution was precisely diluted according to this calculation, thereby providing a stringent test of the sensor’s ultralow-concentration magnetic-bead response capability. In Fig. 5c, the aperture denotes the diameter of the biomolecular detection chamber opening, which determines the effective coupling area. During bead-detection measurements, the sample–sensor distance was fixed at 1.2 mm, and the OFG was operated at 125 kHz. For Fig. 5d–g, external magnetic fields of 0.30, 0.45, and 0.60 Oe were applied.

As shown in Fig. 5c, the relationship between output voltage (ΔV) and MBs concentration was measured for chip apertures of 2, 3, and 4 mm. The results indicate that the 3 mm aperture provides the highest sensitivity to the magnetic beads (Further discussion of the aperture-size effect on magnetic-bead coupling is provided in the Supporting Information). Figure 5d–g shows the output voltage versus concentration curves for MBs with diameters of 100 nm, 200 nm, 500 nm, and 1 μm under varying concentrations. Additional measurements for intermediate bead diameters of 300 and 400 nm were performed under the same concentration range and applied-field conditions, and the results are provided in Fig. S16, and the corresponding particle-number-density and dipolar-scaling analyses are summarized in Tables S13 and S14. The four bead sizes shown in Fig. 5d–g exhibit a characteristic non-monotonic trend, initially rising and then decreasing, but their dynamic ranges and maximum responses differ significantly. Across the different bead diameters, the sensor exhibits a stable quantitative response with size dependent linear regimes. For 100 nm beads, the response is linear between 1 pg mL^−1^–100 ng mL^−1^, whereas 200 nm beads show a linear range of 10 pg mL^−1^–1 μg mL^−1^. The larger 500 nm and 1 μm beads shift to higher linearity windows of 15 pg mL^−1^–1 μg mL^−1^ and 100 pg mL^−1^–10 μg mL^−1^, respectively, consistent with the onset of stronger dipolar interactions at increasing diameters. The 200 nm beads produce the largest change in ΔV (67 mV), indicating an optimal balance between magnetic moment strength and dispersion uniformity. The 100 nm beads, though showing a smaller ΔV (51 mV), remain responsive over a broader concentration range and can still be detected at ultra-low concentrations due to their small size, good dispersion, and weak dipole interaction. In contrast, the 500 nm and 1 μm beads tend to aggregate even at low concentrations (Fig. S12), resulting in earlier signal saturation and a narrower dynamic range. Based on this, we further analyzed the effects of superparamagnetic MBs of different diameters on the output of the custom OFG probe using representative concentration steps (four intervals for quantitative comparison). From a physical perspective, the magnetic moment of a single bead under a bias field H can be expressed in the Langevin form as Eq. (2) [69]:

2 $$m\left(H\right)={M}_{s}{V}_{p}L\left(\frac{{\mu}_{0}{M}_{s}{V}_{\rho }H}{{k}_{B}T}\right)$$

where ${M}_{s}$ is the saturation magnetization, ${V}_{p}$ is the particle volume, and L is the Langevin function. In the low field approximation, the magnetic moment reduces to $m \approx {\chi}_{p}H$, with ${\chi}_{p}\propto {V}_{p}^{2}$. The axial field produced by a single dipole at distance r follows the far field expression ${B}_{dip}\left(r\right)=\frac{{\mu}_{0}}{4\pi }\frac{2m}{{r}^{3}}$ [70]. For multiple beads within the effective coupling volume, the net far-field can be expressed in a form corrected by a structural factor as Eq. (3) [71–73]:

3 $${B}_{beads}\approx \frac{{\mu}_{0}}{4\pi }\frac{2{N}_{eff}m}{{r}^{3}}S\left(0\right)$$

where ${N}_{eff}$ denotes the effective number of coupled beads, m is the average magnetic moment of a single bead under bias, and $S\left(0\right)$ is the structure factor, which reflects spatial correlations among particles; $S\left(0\right)\approx$ 1 for a fully random distribution, while S(0)<1 indicates aggregation or chain formation [73]. The probe’s output is approximately proportional to the bead-induced field: $\Delta {V}_{2m}\approx {S}_{2w}{B}_{beads}$, where ${S}_{w}\propto NA{\mu}_{0}\omega <\frac{{\partial }^{2}M}{{\partial H}^{2}}>t$ denotes the sensitivity of the core under excitation [74, 75]. Based on this relationship, the four concentration regimes observed experimentally can be qualitatively understood as a gradual transition from bead-number-dominated field enhancement to aggregation- and shielding-influenced coupling attenuation, as further discussed in the Supporting Information. In the ultra-low concentration regime (first interval, far left in Fig. 5), MBs are sparse and well separated. The structure factor remains close to unity $S\left(0\right)\approx$ 1, the effective number of coupled beads ${N}_{eff}$ is minimal, corresponding to a statistically diluted low-bead-number detection regime. Consequently, the output increases nearly linearly with concentration. In the low concentration regime (second interval), ${N}_{eff}$ grows significantly while aggregation is still negligible. The product ${N}_{eff}m$ increases rapidly, driving a steep rise in ΔV. In the intermediate regime (third interval, typically near the output maximum), small particles such as the 200 nm beads remain well dispersed, thus $S\left(0\right)$ stays close to unity and the response reached its peak. Larger beads (500 nm and 1 μm), however, experience strong dipole coupling, with interaction energy ${U}_{dd}\approx \frac{{\mu}_{0}{m}^{2}}{4\pi {r}^{3}}$. The corresponding dimensionless parameter $\Lambda =\frac{{U}_{dd}}{kBT}$ scales as $\Lambda \propto {d}^{6}$ [71], increasing rapidly with bead size. Chain formation and aggregation then occur, reducing $S\left(0\right)$ and suppressing far field superposition. As a result, large beads show premature saturation and weaker responses at this stage. In the high concentration regime (fourth interval, right side), aggregation and shielding dominate. Local demagnetization and field blocking emerge within bead clusters, further decreasing $S\left(0\right)$. The net far field contribution declines with increasing concentration, and ΔV drops. These results indicate that, under identical geometry and flow conditions, the 200 nm beads exhibit the strongest output response, as their size offers an optimal trade-off between magnetic moment and dispersion stability. The 100 nm beads, while generating a smaller change in voltage, demonstrate superior sensitivity at low concentrations because their small size and stable suspension prevent aggregation, allowing detectable responses even in dilute samples. By contrast, 500 nm and 1 μm beads, although individually stronger, suffer from aggregation already in the second and third intervals, leading to early peak decay and a narrower dynamic range [15, 76]. For all bead sizes, the maximum output appears under a bias field of 0.45 Oe, where the magnetization slope of the beads best matches the sensor core response, achieving the highest signal-to-noise ratio (Further discussion of the bias-field-dependent magnetic-bead response and the origin of the maximum output at 0.45 Oe is provided in the Supporting Information). These results provide practical guidance: choosing bead diameters that ensure strong magnetization while maintaining uniform dispersion (200 nm in this work) can effectively enhance ΔV and broaden the measurable concentration range. Further improvements can be realized by reducing the bead–sensor distance r, increasing the pickup coil turns N and effective coupling area A, and suppressing aggregation via surface modification and flow control to maintain $S\left(0\right)\approx$ 1. These relationships suggest that the detection limit can be reduced by [77, 78]: ${m}_{min}\approx \frac{2\pi {r}^{3}}{{\mu}_{0}}\frac{{V}_{n}}{{s}_{2\omega }{N}_{{e}_{ff}}s\left(0\right)}$

Further, AFP was selected as a model biomarker for detection. The glass substrate used for magnetic bead immobilization was first functionalized with 3-aminopropyltriethoxysilane (APTES), and the surface modification was characterized by XPS (Fig. 5h, top). As shown, the N 1*s* signal of the unmodified glass is extremely weak, exhibiting only a broad and low intensity background feature around 399–400 eV, indicating that the native surface contains almost no nitrogen species [79]. After treatment with APTES, a strong and nearly symmetric N 1*s* peak emerges at 399–400 eV, confirming the presence of surface amino groups (-NH_2_) from APTES, which provide reactive sites for the subsequent covalent coupling of AFP antigens to the glass surface. The APTES functionalization was further validated by FTIR (Fig. 5h, bottom): the modified glass surface displays pronounced CH₂ stretching (2930 cm^−1^), CH₂ bending (1460 cm^−1^), and enhanced Si–O-Si stretching bands (950–1100 cm^−1^) [80]. The appearance and strengthening of these characteristic bands demonstrate that APTES molecules form a dense siloxane network and introduce organic functional groups on the glass surface, thereby achieving effective silanization and establishing a stable and reproducible interface for subsequent biomolecule immobilization and monodisperse bead distribution [81].

On this basis, AFP antigens were immobilized on the APTES treated glass surface, and specific recognition of AFP as well as magnetic signal readout were realized using immunomagnetic beads (SEM images of the magnetic beads after the immunoreaction are shown in Figs. S13 and S14). The working process of the magnetic bead-based immunoassay for AFP detection is illustrated in Fig. S15. Magnetic response measurements were carried out under an external magnetic field of 0.45 Oe (Fig. 5i). As the AFP concentration increased from 50 fg mL^−1^ to 100 ng mL^−1^, the change in ΔV exhibited a good linear relationship. To further validate the analytical reliability of the AFP immunomagnetic assay, blank-control, selectivity, interference, and repeatability tests were performed. Validation in serum matrices will be pursued in future work. The results, including blank-background correction and interference evaluation using BSA and PSA, are provided in the Supporting Information. Together with the broad linear range, this detection limit confirms the capability of the present OFG platform to resolve trace AFP signals in an immunomagnetic assay format. As summarized in Table S15, the obtained performance is competitive among representative AFP magnetic biosensors, reflecting the combined contributions of low-frequency sensor noise, bead labeling efficiency, bead-sensor coupling, and signal readout configuration. The present proof-of-concept results demonstrate that the OFG platform can resolve trace AFP signals in an immunomagnetic assay format. With an appropriate dilution strategy, the detectable concentration range may be further extended to higher levels. Since serum-matrix validation has not yet been performed in this work, future studies will focus on serum-based testing, anti-interference optimization in complex matrices, and clinical-sample evaluation. These efforts will further assess the potential of this platform for clinical-oriented AFP biosensing.

## Conclusions

This study systematically investigates low-noise core design for OFGs and their application in ultralow concentration MBs detection. By adjusting electrochemical deposition current density and annealing parameters, the magnetic hysteresis, domain structures, and their influence on noise performance of composite cores were comprehensively analyzed. By integrating material engineering and circuit optimization, including nanocrystalline fabrication, domain structure control, and closed-loop feedback readout, the sensor achieved a system-level input-referred equivalent magnetic noise of 8 pT/√Hz at 1 Hz. Biosensing tests revealed four characteristic response regimes, with 200 nm MBs showing the largest ΔV under identical conditions. The 100 nm MBs, although yielding a smaller voltage change ΔV than larger beads, exhibit the highest signal-to-noise ratio and reliable detection performance over the ultralow-concentration range of 1 pg mL^−1^ -100 ng mL^−1^, owing to their smaller size and superior dispersion. When AFP is employed as a biomarker in an immunomagnetic bead assay, the sensor output displays a well-defined linear dependence on AFP concentration from 50 fg mL^−1^ to 100 ng mL^−1^, with a detection limit as low as 50 fg mL^−1^, demonstrating competitive sensitivity for AFP immunomagnetic detection using the present OFG-based platform. Theoretical analysis indicates that the detection sensitivity is jointly governed by the structural parameters between the beads and the sensor, the magnetic moment of individual beads, and related factors, among which bead dispersion and an optimized bias field are identified as key determinants for performance enhancement. Collectively, these results establish a consistent structure-magnetism-noise correlation among structure, magnetic behavior, and noise in CoP/Ag-based OFGs, suggesting that amorphous-nanocrystalline structural ordering contributes to reduced magnetic noise. This work further provides a practical strategy for high-sensitivity biosensing over a wide dynamic range.

## Supplementary Information

Below is the link to the electronic supplementary material.

Supplementary file1 (DOCX 24256 KB)
