# Supplementary material for: Ultralow Noise Orthogonal Fluxgates Enabling Weak Magnetic Field and Biomolecular Detection
Source: Nanomicro Lett. 2026 Aug 3;19:19. doi: 10.1007/s40820-026-02317-2 (PMC13433690; doi:10.1007/s40820-026-02317-2)
Supplement: Supplementary file 1 [file 40820_2026_2317_MOESM1_ESM.docx]

Supporting Information for

**Ultralow Noise Orthogonal Fluxgates Enabling Weak Magnetic Field and Biomolecular Detection**

Xiaofeng Pu^1^, Zhijun Sheng^1^, Zhoulu Yu^1^, Huaidong Li^1^, Xintao Wei^1^, Tao Yang^1^, Qingfang Liu^1^, Guozhi Chai^1^, Xiaolei Liang^2^, Daqiang Gao*^1^

^1^ Key Laboratory of Magnetism and Magnetic Functional Materials (Lanzhou University), Ministry of Education, Lanzhou 730000, P. R. China

^2^ Department of Obstetrics and Gynecology, Key Laboratory for Gynecologic Oncology Gansu Province, The First Hospital of Lanzhou University, Lanzhou 730000, P. R. China

*Corresponding author. E-mail: [gaodq@lzu.edu.cn](mailto:gaodq@lzu.edu.cn) (Daqiang Gao)

**Supplementary Text**

**S1 Details of micromagnetic simulation**

Micromagnetic simulations in this work were performed using the GPU-accelerated software Mumax^3^ [S1, S2]. In the simulation, the fourth-order Runge-Kutta method is used to solve the Landau-Lifshitz-Gilbert (LLG) equation numerically. The temperature is set to 0 K to avoid interference from the thermal disturbance. Regarding the size limitations inherent to micromagnetic simulations, the target structure was proportionally scaled down to the nanometer regime. While this scaling approach is known to increase the coercive field-an expected artifact of reduced size-it preserves the fundamental magnetic behavior and qualitative trends of the original system, ensuring the validity of the simulation results. The micromagnetic parameters used in the simulation are as follows: the ferromagnet is a CoP tube with an inner diameter of 100 nm and an outer diameter of 125 nm, whose saturation magnetization M_S_ = 2.66×10^6^ A/m, exchange stiffness constant A = 3×10^-11^ J/m [S3]. The magnetocrystalline anisotropy constant K_u_ is differentiated by phase: 4.5×10^5^ J/m^3^ for the Type C phase and 1×10^4^ J/m^3^ for the Type A phase. In the micromagnetic simulation, Type A and Type C were used to represent the low-order amorphous-like region and the highly ordered nanocrystalline region, respectively. Type B corresponds to an intermediate short-range ordered structure between these two states, and was therefore not assigned as an independent magnetic phase in the simulation.

To reduce the computational cost of full-scale micromagnetic simulations, the tubular CoP structure was geometrically scaled down to the nanoscale in this work. Therefore, the simulations were not intended to quantitatively reproduce the absolute coercive field, switching field, or noise level of the real magnetic core. Instead, they were used to reveal the relative changes in magnetization-rotation behavior induced by structural ordering.

From a micromagnetic perspective, the influence of structural ordering on magnetization rotation is mainly governed by the competition among local exchange coupling, magnetic anisotropy, and magnetostatic interactions. Thus, although dimensional scaling may alter the absolute field values, the qualitative trends remain physically meaningful when the disordered and ordered models are compared using the same scaled geometry, boundary conditions, and excitation conditions [S4, S5]. More importantly, the two simulation models before and after introducing the dual-phase structure used the same scaled geometry, boundary conditions, and excitation conditions. Therefore, the comparison focuses on the relative changes in magnetization-rotation uniformity and angular mismatch between neighboring regions, rather than on absolute magnetic-field values.

In the disordered model, the magnetic moments exhibit segmented and partially decoupled responses under excitation. After introducing the amorphous-nanocrystalline dual-phase structure and enhancing lattice ordering (Fig. 2d), the angular mismatch between neighboring regions decreases [S6] and the magnetization process evolves into a more continuous and coordinated rotational state. This simulation trend is consistent with the experimental results. TEM and ACF analyses show an increased fraction of ordered regions after annealing. The dynamic hysteresis loops and noise spectra further reveal reduced magnetic loss and lower 1 Hz noise. Therefore, the simulations are mainly used to support the mechanistic link that structural ordering promotes coordinated magnetization rotation and thereby contributes to low-frequency noise suppression.

**S2 Technical Positioning of the OFG**

To further clarify the technical position of the present OFG among advanced magnetic sensing technologies, we added a quantitative comparison with representative OPM, TMR, ME, SQUID, and fluxgate sensors in Table S1. Since these sensors rely on different magnetic-to-electrical transduction mechanisms, their voltage sensitivities cannot be directly compared under a unified standard. Therefore, system-level input-referred equivalent magnetic noise or field detectivity was used as the main comparison metric.

OPM and SQUID sensors can reach fT/√Hz or even sub-fT/√Hz noise levels, but they usually require optical or cryogenic systems, magnetic shielding, or near zero field operation. TMR sensors offer excellent miniaturization and array compatibility, but their low-frequency performance is often limited by 1/*f* noise. ME sensors can achieve pT/√Hz-level detectivity at room temperature, but their performance is strongly affected by mechanical resonance and vibration noise. In this context, the CoP/Ag-based OFG achieves a system-level input-referred equivalent magnetic noise of 8 pT/√Hz at 1 Hz and a sensitivity of 153.8 kV/T under room-temperature closed-loop operation, demonstrating its overall advantages for low-frequency weak field sensing, miniaturization, and magnetic-bead-based biosensing.

**Table S1** Comparison of recent representative magnetic sensing technologies

| Sensor technology | Representative magnetic noise | Typical conditions | Main features and limitations | Refs. |
| --- | --- | --- | --- | --- |
| SQUID | Sub-fT/√Hz white-noise level | Cryogenic operation; usually shielded | Highest sensitivity, but requires low-temperature system and high-cost setup | [S7] |
| OPM | 30 fT/√Hz for recent closed-loop SERF OPMs | Alkali-vapor cell; laser pumping; near-zero or compensated field | Cryogen-free fT-level detection, but needs optical system, heating and field compensation | [S8] |
| TMR | sub-pT to pT/√Hz at 1 Hz in optimized devices | Room temperature; MTJ bridge; often with modulation or flux concentrators | Highly miniaturized and array-compatible, but limited by low-frequency 1/f noise and offset drift | [S9] |
| ME sensor | pT/√Hz to sub-pT/√Hz at 1 Hz | Room temperature; magnetostrictive/piezoelectric coupling | Room-temperature pT-level detection, but sensitive to resonance, vibration and bias conditions | [S10] |
| Fluxgate/OFG | 5–10 pT/√Hz at 1 Hz in recent compact systems | Room temperature; magnetic-core excitation and pickup coil | Robust low-frequency detection and wide dynamic range, but requires core excitation and magnetic-core optimization | [S11] |
| This work | 8 pT/√Hz at 1 Hz; | Room temperature; closed-loop OFG; compact CoP/Ag core | pT-level low-frequency noise, simple structure, low-cost fabrication and good compatibility with immunomagnetic bead detection | / |

**S3 Functional description of the closed-loop OFG signal-conditioning circuit**

Fig. 1c shows the closed-loop signal-conditioning circuit and photograph of the fabricated OFG sensor. The circuit consists mainly of a clock-generation module, an excitation-driving module, a CoP/Ag composite-core sensor probe, a pick-up signal amplification and synchronous demodulation module, and a closed-loop feedback module.

In the excitation section, the 74HC00 logic gate and RC network are used to generate a stable square-wave clock signal, which provides the reference excitation for the OFG. The frequency-divider resistor is used to set the excitation-frequency condition, whereas the series current-limiting resistor R regulates the excitation-current amplitude and improves driving stability. The square-wave signal is then amplified by the SQ4532 power MOSFET to drive the excitation coil wound around the CoP/Ag composite core. This excitation current generates a periodic circumferential magnetic field in the core, thereby periodically modulating the magnetization state required for orthogonal fluxgate operation.

In the sensor probe, the CoP/Ag composite core acts as the magnetic sensing element, while the pick-up coil detects the voltage induced by the field-modulated magnetization process (The orange line in the probe indicates the position of the glass tube). When an external axial magnetic field is applied, the axial magnetization component of the core is modulated by the circumferential excitation field, producing a pick-up voltage related to the external magnetic field. Because this induced signal is weak and may contain excitation feedthrough, harmonic components, and low-frequency noise, further low-noise amplification and phase-sensitive detection are required.

The pick-up signal is first amplified by the ADA4522 precision low-noise amplifier to increase the signal amplitude while minimizing the degradation of the signal-to-noise ratio. The amplified signal is then fed into the ADG620 synchronous demodulator, which uses the clock signal derived from the same excitation reference to perform phase-sensitive detection. This process extracts the magnetic-field-dependent signal component and suppresses asynchronous noise and high-frequency interference. The demodulated signal is further amplified and filtered by the OP27 and ADA4622 stages, yielding a DC output voltage that is approximately linear with the applied magnetic field.

To improve linearity and zero-field stability, a closed-loop feedback branch based on the AD8009 is incorporated. Part of the output signal is fed back to the sensor probe to generate a compensation field, keeping the magnetic core close to a stable operating point. This closed-loop operation suppresses zero drift, extends the linear response range, and reduces the influence of environmental perturbations and core nonlinearity on the output. Therefore, the circuit combines stable excitation, low-noise amplification, synchronous demodulation, and closed-loop feedback to effectively read out the low-noise response of the CoP/Ag composite core.

***S4.1* Elemental composition analysis**

For each sample, five representative regions within the CoP coating were selected for TEM-EDS area analysis, avoiding the Ag substrate, the CoP/Ag interfacial region, and obvious pores or contamination. The Co/P atomic ratios obtained from different regions were consistent, and the averaged compositions are summarized in Tables S2-S4.

**Fig. S1 a** Selected-area compositional analysis of the CoP layer. **b** elemental mapping of the CoP/Ag magnetic core.

**Table S2** CoP element ratio at 1.2A/dm^2^ current density

| Element | At. No. | Mass Norm. [%] | Atom [%] | abs.error [%]  [mass%] (1 σ) |
| --- | --- | --- | --- | --- |
| P | 15 | 6.93 | 12.41 | 0.32 |
| Co | 27 | 93.07 | 87.59 | 2.37 |
|  |  | 100.00 | 100.00 |  |

**Table S3** CoP element ratio at 1.0 A/dm^2^ current density

| Element | At. No. | Mass Norm. [%] | Atom [%] | abs.error[%]  [mass%] (1 σ) |
| --- | --- | --- | --- | --- |
| P | 15 | 8.03 | 14.24 | 0.37 |
| Co | 27 | 91.97 | 85.76 | 2.34 |
|  |  | 100.00 | 100.00 |  |

**Table S4** CoP element ratio at 0.8 A/dm^2^ current density

| Element | At. No. | Mass Norm. [%] | Atom [%] | abs.error[%]  [mass%] (1 σ) |
| --- | --- | --- | --- | --- |
| P | 15 | 10.71 | 18.58 | 0.49 |
| Co | 27 | 89.29 | 81.42 | 2.27 |
|  |  | 100.00 | 100.00 |  |

To clarify the simulation design, field condition, and intended interpretation of Figs. 2c-d, the key settings are summarized in Table S5.

**Table S5** Key settings and interpretation of the micromagnetic simulations

| Item | Fig. 2c | Fig. 2d | Purpose |
| --- | --- | --- | --- |
| Structural representation | Disordered amorphous-like state | Amorphous-nanocrystalline ordered state | Compare the effect of local structural ordering |
| Geometry | CoP tube, 100 nm inner diameter and 125 nm outer diameter | Same | Keep geometric conditions identical |
| Magnetic parameters | *M*_s_=2.66×10 ^6^A/m, A=3.0×10^-11^ J/m | Same | Keep basic magnetic parameters identical |
| Effective anisotropy | *K*_u_=1.0×10 4 J/m³ | *K*_u_=4.5×105 J/m³ | Represent different local structural-order states |
| External axial DC field | None | None | Show relaxed local magnetization configurations |
| Figure meaning | More segmented local moment arrangement | More continuous local moment arrangement | Qualitative comparison of magnetization continuity |

It should be clarified that Figs. 2c-d visualize the spatial distribution of the local magnetization vector **M**, rather than magnetic flux lines **B**. In the micromagnetic calculation, the demagnetizing field is solved self-consistently from the magnetostatic equations, and the resulting magnetic induction satisfies **∇⋅B**=0. Therefore, the simulated magnetization configurations are consistent with Maxwell’s equations.

***S4.2* Reference annealing temperatures for Co****-P structural evolution**

To help interpret the structural evolution of the CoP layer after annealing, we further referred to previous studies on the thermal treatment of electrodeposited Co-P coatings. Existing reports show that electrodeposited amorphous Co-P coatings readily undergo structural relaxation, local ordering, and crystallization under suitable heat treatment. Eliaz et al. reported that the crystallization temperature of electrodeposited amorphous Co-P coatings is approximately 284 °C, with the main phase transformation occurring in the range of 300-400 °C[S3]. Bera et al. further showed by XPS that oxidized Co species may exist on the Co-P surface, supporting the need to analyze oxygen-related surface states [S12]. In this work, the annealed CoP layer exhibits nanocrystalline clusters, SAED diffraction rings, and an XRD pattern still dominated by a broad amorphous background. These features indicate that the annealing process mainly induces local structural ordering and partial nanocrystallization, rather than complete bulk crystallization. This behavior is consistent with the thermally induced amorphous-to-nanocrystalline transition reported for Co–P systems in the literature.

**Fig. S2 a-b** SEM images of the cross section and surface of the composite structure core. **c** The TEM image and selected area electron diffraction of unannealed CoP/Ag. **d** The TEM images and selected area electron diffraction of CoP/Ag after annealing.

**Fig. S3** Annealed and unannealed core XRD data.

***S4.3* XPS analysis of surface oxygen evolution before and after current-assisted annealing**

To examine whether current-assisted annealing changes the surface oxidation state of the CoP coating, we performed XPS survey semi-quantitative analysis on unannealed and annealed samples, as shown in Fig.S4. High-resolution Co 2p, P 2p, and O 1s spectra were also collected. The surface O content decreases from 28.85 at.% before annealing to 23.86 at.% after annealing. Meanwhile, the Co/P atomic ratio remains nearly unchanged, changing only from 1.28 to 1.29. These results indicate that current-assisted annealing in air does not increase the surface oxygen content of the CoP coating or cause an obvious change in its relative Co/P composition. The high-resolution Co 2p spectra show that both samples retain the Co 2p_3/2_, Co 2p_1/2_, and satellite features, confirming the persistence of Co-related surface chemical states after annealing. In the P 2p spectra, phosphide-related P peaks and P-O components are observed before and after annealing, indicating the presence of oxygen-related surface species on the CoP coating. The O 1s spectra further confirm oxygen-containing species on both sample surfaces. Although the relative contributions of the high-resolution components vary after annealing, the Co/P ratio remains essentially unchanged. Therefore, together with the survey semi-quantitative results, these data indicate that the present current-assisted annealing treatment does not induce pronounced additional surface oxidation.

**Fig. S4** High-resolution XPS spectra of unannealed and annealed CoP coatings: **a** Co 2p. **b** P 2p, and **c** O 1s. **d** XPS survey spectra.

***S4.4* Semi-quantitative ACF criteria for structural classification**

To provide a semi-quantitative criterion for distinguishing the Type-A, Type-B, and Type-C regions, local autocorrelation function (ACF) analysis was performed on representative HRTEM regions [S13]. The original HRTEM images were first calibrated in DigitalMicrograph (DM), and regions of interest (ROIs) with the same size were selected from the CoP coating region. The selected ROIs were then subjected to local ACF analysis, and uncompressed grayscale images were exported. The grayscale intensity extraction and profile analysis of the ACF images were performed using ImageJ, while the subsequent calculations and curve processing were carried out using Origin.

The ACF images were first converted into 8-bit grayscale images, with an intensity range of 0-255. The brightest peak at the center of each ACF image was defined as the central autocorrelation peak, and its grayscale intensity was denoted as *I*_center_ . Since the central peaks in the representative Type-A, Type-B, and Type-C regions were close to saturation, *I*_center_ was taken as: *I*_center_≈255

The background intensity *I*_bg_ was determined from regions far from the central and periodic peaks. Specifically, background regions were selected from the four corners of each ACF image, and the median grayscale value of these regions was taken as *I*_bg_. A grayscale line profile passing through the central peak was then extracted along the main periodic direction of the ACF image. The grayscale value of the first distinguishable structural correlation peak outside the central peak was defined as *I*_first_.

The normalized first non-central ACF peak intensity is defined as:

$$C_{ACF}=\frac{I_{first}-I_{bg}}{I_{center}-I_{bg}}$$

where *C*_ACF_ is used to describe the strength of the local periodic signal. A larger *C*_ACF_ indicates stronger local periodicity in the analyzed region.

The correlation length *L*_corr_ is defined as the distance at which the background-corrected ACF signal decays to 1/*e* of the first non-central ACF peak intensity. This parameter describes how far the ACF periodic signal persists in space, corresponding to the extension length of local periodic ordering. The detailed calculation process is described below using Type-A as an example.

***S4.5* Calculation for the Type-A region**

For the Type-A region, the periodicity outside the central peak in the ACF image is weak, the stripe continuity is poor, and the signal becomes rapidly diffuse away from the center. Therefore, when extracting *I*_first_, the bright ring near the central peak caused by the extension of the saturated central peak was not regarded as an effective structural periodic peak. Instead, the first weak but distinguishable structural correlation peak outside the central peak was selected.

The grayscale profile was extracted using ImageJ, giving:

*I*_center_≈255, *I*_bg_≈35, *I*_first_≈85

Here, *I*_center_ was obtained from the central bright peak of the ACF image, *I*_bg_ was obtained from the median grayscale value of the four corner background regions, and *I*_first_ was obtained from the grayscale value of the first weak structural correlation peak outside the central peak.

Substituting these values into the equation gives:

$C_{ACF}^{Type-A}$≈0.23

Therefore, the normalized first non-central ACF peak intensity of the Type-A region is: $C_{ACF}^{Type-A}$≈0.23

The correlation length *L*_corr_ was then calculated. The background-corrected first peak intensity is:

*A*_1_=*I*_first_−*I*_bg_=85−35=50

The corresponding 1/*e* decay intensity is:

A*_1/e_*=50/2.718≈18

From the ACF grayscale profile, the periodic peaks in the Type-A region decay rapidly, and the background-corrected signal approaches the A*_1/e_* level at approximately *r*=18-22 pixels. Therefore,

$L_{corr}^{Type-A}$≈20 pixels

This result indicates that the Type-A region has only weak local periodicity and a short correlation length, corresponding to a disordered or nearly amorphous structure.

The values calculated for the other types are summarized in the table below:

**Table S6** Quantitative comparison of Type-A, Type-B, and Type-C regions

| Type | *I*_center_ | *I*_bg_ | *I*_first_ | *C*_ACF_ | *L*_corr_ |
| --- | --- | --- | --- | --- | --- |
| Type-A | 255 | 35 | 85 | 0.23 | 20 pixels |
| Type-B | 255 | 40 | 130 | 0.42 | 29 pixels |
| Type-C | 255 | 42 | 172 | 0.61 | 45 pixels |

As shown by the above results, both *C*_ACF_ and *L*_corr_ gradually increase from Type-A to Type-C. The Type-A region shows only weak local correlation peaks and a short correlation length, corresponding to a disordered or nearly amorphous structure. The Type-B region exhibits ACF peaks of moderate intensity and short-range periodicity, and can therefore be assigned to a short-range ordered region. The Type-C region shows stronger ACF peaks and a longer correlation length, indicating clearer local periodicity and corresponding to an ordered nanocrystalline region.

Based on the *C*_ACF_ and *L*_corr_ values of the representative Type-A, Type-B, and Type-C regions, the midpoint values between adjacent types were used as semi-quantitative classification thresholds.

For *C*_ACF_:

$C_{ACF,A/B}$=$\frac{0.23+0.42}{2}==0.325\approx0.33$

$C_{ACF,B/C}$=$\frac{0.42+0.61}{2}=0.515\approx0.52$

For *L*_corr_:

$L_{corr,A/B}$=$\frac{20+29}{2}=24.5\approx25$

$L_{corr,B/C}$=$\frac{29+45}{2}=37$

Therefore, the following semi-quantitative evaluation criteria were adopted:

**Table S7** Recommended evaluation criteria

| Type | *C*_ACF_ *criterion* | *L*_corr_ *criterion* | Structural feature |
| --- | --- | --- | --- |
| Type-A | *C*_ACF_ <0.33 | *L*_corr_<25 pixels | Disordered, nearly amorphous region |
| Type-B | 0.33≤*C*_ACF_<0.52 | 25≤Lcorr<37 pixels | Short-range ordered region |
| Type-C | *C*_ACF_≥0.52 | Lcorr≥37 pixels | Local nanocrystalline, more ordered region |

When the assignments based on *C*_ACF_ and *L*_corr_ showed slight differences, the corresponding local FFT patterns were further used as auxiliary criteria. Regions with diffuse FFT features and no clear periodic peaks were assigned to Type-A. Regions with weak periodic FFT features were assigned to Type-B. Regions with clear FFT spots or obvious periodic features were assigned to Type-C. Therefore, the classification of Type-A, Type-B, and Type-C in this work was based on the combined evaluation of *C*_ACF_, *L*_corr_, and local FFT features.

***S5.1* Schematic of the dynamic circumferential hysteresis-loop measurement**

The schematic diagram of the dynamic circumferential hysteresis-loop measurement for the magnetic core is shown in Fig. S5. In the test system, a signal generator (Keysight 81160A) was used to generate sinusoidal excitation signals with different frequencies and amplitudes. This signal was applied to the composite-wire magnetic core as the excitation source and was simultaneously input into the oscilloscope as a reference signal. The induced signal generated by the magnetic core under the alternating excitation current was acquired in real time by the oscilloscope, forming a closed excitation and detection loop together with the excitation signal. Subsequently, the acquired voltage and current signals were synchronously processed and calculated using a LabVIEW program to reconstruct the dynamic circumferential hysteresis loops of the magnetic core. This method enables the dynamic magnetization behavior of the magnetic core under different excitation conditions to be obtained, providing experimental evidence for analyzing the high-frequency magnetic response of the core and its influence on sensor performance.

**Fig. S5** Schematic of the dynamic circumferential hysteresis-loop measurement.

**Fig. S6** Circumferential dynamic hysteresis loops of different electrochemical deposition conditions under 10 kHz, 0 mA bias, amplitude varying excitation conditions; the annealed 1.0 A/dm2 sample was treated at 1.8 A for 15 min. **a-c** Effect of deposition time for samples electrodeposited at 1.0 A/dm² and annealed at 1.8 A for 15 min: a 6500 s, b 7500 s, and c 8500 s. **d-f** Effect of annealing current for samples electrodeposited at 1.0 A/dm² for 7500 s and annealed for 15 min: d 1.6 A, e 1.8 A, and f 2.1 A.

***S5.2* Comparison of surface circumferential domain amplitude and density**

To further quantitatively compare the surface circumferential domain characteristics of different samples, the domain amplitude and density were analyzed from the magnetic-domain images in Fig. 3i-l, as shown in Fig. S7. The line-profile characteristics, apparent domain width, width dispersion, dynamic hysteresis-loop area, and 1 Hz noise are summarized in Table S8. Here, the domain-signal amplitude reflects the variation in magnetic-domain contrast, while the domain density was estimated from the number of characteristic domain peaks within a representative 10 μm region. These parameters were used to describe the relative clarity, regularity, and compactness of the circumferential domain arrangement.

The unannealed 1.0 A/dm^2^ sample shows broad and nonperiodic ripple-like domains. This indicates stronger residual stress, defect pinning, and fluctuations in the local anisotropy field. After annealing, the 1.0 A/dm^2^ sample develops clearer stripe-like domains. Its line-scan profile exhibits a quasi-periodic peak-valley distribution. The apparent domain width is approximately 0.8 μm, with a lower domain-width dispersion. These results indicate that current-assisted annealing induces amorphous–nanocrystalline structural ordering and residual-stress relaxation. As a result, the local effective anisotropy field becomes more uniform and the domain arrangement becomes more regular. Consistently, the circumferential dynamic hysteresis-loop area decreases from 4.34×10^−6^ to 2.52×10^−6^ Wb/mA, and the lowest 1 Hz noise of 8 pT/√Hz is obtained. This suggests that the ripple-to-stripe transition is closely associated with reduced irreversible magnetization and lower dynamic magnetic loss.

In contrast, the 1.2 A/dm^2^ sample shows irregular short-range fluctuations in the line-scan profile. A well-defined apparent domain width cannot be extracted, indicating a blurred and disordered domain structure. This behavior mainly arises from the high electrodeposition current density, which accelerates CoP layer growth and introduces stronger compositional fluctuation, residual-stress accumulation, and reduced amorphous-structure stability. These effects broaden the distribution of local anisotropy and enhance domain-wall pinning. Consequently, this sample exhibits higher dynamic magnetic loss and higher 1 Hz noise. Overall, the quantitative results in Table S8 further support the correlation among a more regular circumferential domain structure, lower dynamic magnetic loss, and reduced low-frequency noise in the optimally annealed CoP/Ag core.

**Fig. S7** Comparison of the surface circumferential domain amplitude and density for the 1.0 A/dm^2^ CoP/Ag core before and after annealing at 1.8 A for 15 min, and for samples prepared under different deposition current densities.

**Table S8** Comparison of the surface circumferential domain amplitude and density for the 1.0 A/dm^2^ CoP/Ag core before and after annealing at 1.8 A for 15 min, and for samples prepared under different deposition current densities

| Sample | Domain morphology | Line-profile feature | Apparent domain width *w*_app_ | *CV* | Dynamic hysteresis loop area/10^−6^ Wb/mA | 1 Hz noise / pT/√Hz | Physical implication |
| --- | --- | --- | --- | --- | --- | --- | --- |
| Unannealed 1.0 A/dm^2^ | Ripple-like domains | Broad, nonperiodic fluctuations | broad distribution | High | 4.34 | Higher than annealed sample | Nonuniform local anisotropy distribution, strong residual stress, and pronounced domain-wall pinning |
| Annealed-1.0 A/dm^2^ | Regular stripe-like domains | Quasi-periodic oscillations with clear peaks and valleys | 0.8 μm | Low, 0.10–0.15 | 2.52 | 8 | More uniform effective anisotropy distribution, enhanced cooperative magnetization rotation, and reduced dynamic magnetic loss |
| 0.8 A/dm^2^ | Coarse, distorted domains | Large peak–valley fluctuations with weak periodicity | 0.9–1.0 μm | Moderate | 3.12 | 61 | Insufficient structural ordering, defect-induced domain-wall pinning, and nonuniform magnetization |
| 1.2 A/dm^2^ | Blurry, disordered domains | Irregular short-range fluctuations with unstable peaks and valleys | fragmented | High | 3.00 | 126 | Composition fluctuation, residual stress, enhanced local-anisotropy dispersion, and severe domain-wall pinning |

Note: *w*_app_ denotes the apparent domain width extracted from the magnetic-domain line-profile. When the peak-to-peak distance *d*_peak−peak_ is regarded as one complete magnetic-domain contrast period, *w*_app_≈*d*_peak−peak_/2. This parameter is used to compare the relative domain scale and ordering among different samples, and should not be interpreted as an absolute domain width directly bounded by domain walls. The coefficient of variation is defined as *CV*=*σ*_w_/*w*_app_, which describes the dispersion of the apparent domain-width distribution.

***S5.3* Quantitative correlation among microstructural ordering, dynamic magnetic loss, and low-frequency noise**

Table S9 summarizes the quantitative relationship among microstructural evolution, dynamic magnetic loss, and low-frequency magnetic noise in the CoP/Ag composite core. After current-assisted annealing, the fraction of ordered Type-C regions increases from 7.9% to 47.4%, indicating a marked enhancement in local structural ordering. Meanwhile, the area of the circumferential dynamic hysteresis loop decreases from 4.34×10^−6^ to 2.52×10^−6^ Wb/mA, corresponding to a reduction of approximately 41.9%. This result indicates a clear suppression of irreversible magnetization processes and dynamic magnetic loss. Consistently, the optimized sample exhibits the lowest system-level input-referred equivalent magnetic noise of 8 pT/√Hz at 1 Hz, which is much lower than the values of 61 and 126 pT/√Hz for the 0.8 and 1.2 A/dm² samples, respectively. These results show that improved local structural ordering, better domain regularity, and reduced dynamic magnetic loss are accompanied by lower low-frequency magnetic noise. Thus, a clearer quantitative correlation is established between the microstructural characterization and the noise behavior.

**Table S9** Quantitative evidence supporting the correlation among microstructural ordering, dynamic magnetic loss, and low-frequency magnetic noise in CoP/Ag composite cores

| Category | Parameter | Comparison basis | Unannealed/non-optimized state | Annealed/optimized state | Variation |
| --- | --- | --- | --- | --- | --- |
| Microstructural ordering | Fraction of Type-C ordered regions | Same 1.0 A/dm^2^ sample before and after annealing | Unannealed: 7.9% | Annealed at 1.8 A: 47.4% | +39.5 percentage points; approximately 6.0 times higher |
| Dynamic magnetic loss | Circumferential dynamic hysteresis loop area / Wb·mA^-1^ | Same 1.0 A/dm2 sample before and after annealing | Unannealed: 4.34 × 10^-6^ | Annealed at 1.8 A: 2.52 × 10^-6^ | Decreased by approximately 41.9% |
| Soft magnetic response | Initial susceptibility/Wb·mA^-1^ | Different deposition conditions | — | Optimized 1.0 A/dm^2^ sample: 7.69 × 10^-7^ | — |
| noise | system-level input-referred equivalent magnetic noise at 1 Hz/pT·Hz^-1/2^ | Different deposition current densities | 0.8 A/dm^2^ sample: 61 | Optimized 1.0 A/dm^2^ sample: 8 | Decreased by approximately 86.9% |
| noise | system-level input-referred equivalent magnetic noise at 1 Hz/pT·Hz^-1/2^ | Different deposition current densities | 1.2 A/dm^2^ sample: 126 | Optimized 1.0 A/dm^2^ sample: 8 | Decreased by approximately 93.7% |
| Magnetic domain structure | Domain morphology | Representative domain states | Irregular ripple-like, coarse distorted, or blurred disordered domains | More regular stripe-like domains in the optimized annealed 1.0 A/dm^2^ sample | Qualitative improvement |

**Table S10** Frequency response data of the OFG sensor

| Frequency (Hz) | Output voltage (V) | Normalized response | Attenuation (dB) |
| --- | --- | --- | --- |
| 0.1 | 2.560 | 1.0000 | 0.000 |
| 0.5 | 2.560 | 0.9999 | -0.001 |
| 1 | 2.559 | 0.9998 | -0.002 |
| 5 | 2.545 | 0.9941 | -0.051 |
| 10 | 2.502 | 0.9772 | -0.201 |
| 15 | 2.434 | 0.9507 | -0.439 |
| 20 | 2.348 | 0.9171 | -0.752 |
| 25 | 2.249 | 0.8786 | -1.124 |
| 30 | 2.144 | 0.8376 | -1.539 |
| 35 | 2.03 | 0.7958 | -1.984 |
| 40 | 1.932 | 0.7546 | -2.446 |
| 42 | 1.891 | 0.7385 | -2.633 |
| 43 | 1.870 | 0.7305 | -2.727 |
| 44 | 1.850 | 0.7226 | -2.822 |
| 45 | 1.830 | 0.7148 | -2.916 |
| **46** | **1.810** | **0.7071** | **-3.010** |
| 47 | 1.791 | 0.6995 | -3.105 |

***S6.1* Noise-spectrum averaging, magnetic-noise conversion, and reproducibility analysis**

The voltage noise spectrum shown in Fig. 4 was obtained by averaging 20 independently acquired spectra under identical shielding, excitation, and circuit conditions, rather than by selecting the lowest-noise result from a single measurement. Individual spectra typically exhibit pronounced random fluctuations and spike-like features, whereas the averaged spectrum shows fewer spectral spikes and a smoother profile. Based on the calibrated sensitivity of 153.8 kV/T, the averaged voltage noise density of 1.27 μV/√Hz at 1 Hz corresponds to a system-level input-referred equivalent magnetic noise of approximately 8 pT/√Hz in the manuscript. In addition, repeated measurements performed during parameter optimization and final verification consistently reproduced a 1 Hz noise level close to this value, indicating that the reported noise floor reflects the reproducible averaged performance of the optimized OFG.

***S6.2* System-level noise and circuit-contribution analysis of the closed-loop OFG**

The reported 8 pT/√Hz is defined as the closed-loop system-level input-referred magnetic noise of the complete OFG platform. This value includes the combined contributions of the magnetic core, pickup coil, excitation circuit, synchronous demodulation circuit, amplification stage, feedback loop, and data-acquisition chain. No electronic background noise was subtracted. Therefore, this result reflects the practical noise floor of the complete sensor system.

It should be noted that, in a closed-loop OFG, the circuit contribution cannot be treated as an independent constant background that can be directly subtracted from the total sensor noise. The excitation circuit, magnetic core, pickup coil, synchronous demodulation, amplification stage, and feedback loop are coupled through the closed-loop magneto-electric transduction path. Shorting the input or replacing the pickup coil with a dummy load would change the input impedance, excitation coupling, demodulation residuals, feedback operating point, and noise-transfer function. Therefore, such pure circuit tests can serve as diagnostic references for the electronic readout chain, but they do not represent the physical noise state of the OFG under actual operating conditions and cannot be rigorously subtracted from the full-system noise.

To improve the reproducibility of the noise results, the noise spectrum shown in Fig. 4c was obtained by averaging 20 independently acquired spectra under identical shielding, excitation, feedback, amplification, and acquisition conditions. Individual spectra usually contain random fluctuations and occasional spike-like features. After averaging, the spectrum becomes smoother and the random spikes are markedly reduced. The same sensitivity calibration and noise-conversion procedure were applied to all repeated measurements and comparisons among cores prepared under different processing conditions.

***S6.3* Origin of increased 1 Hz noise under high-frequency excitation**

The increase in 1 Hz noise under high-frequency excitation is mainly attributed to the combined effects of enhanced dynamic magnetic loss in the core and nonideal circuit behavior. For the CoP/Ag composite core, increasing the excitation frequency raises d*B*/d*t*, which strengthens eddy-current loss and field nonuniformity associated with the skin effect. These effects can increase irreversible magnetization processes and low-frequency magnetization fluctuations.

At the same time, high-frequency square-wave excitation introduces stronger switching transients, parasitic coupling, and phase errors in synchronous demodulation. As a result, carrier-related noise may be down-converted into the low-frequency output during demodulation. Therefore, the higher noise observed at 500 kHz should not be attributed to a single factor. Instead, it results from the combined influence of increased magnetic-core loss and nonideal excitation/demodulation circuit effects.

Among the tested conditions, 125 kHz provides a better balance between stable circumferential magnetization and reduced high-frequency loss and crosstalk, thereby yielding the lowest 1 Hz noise.

***S6.4* Circuit contribution in the closed-loop OFG**

It should be noted that the noise spectrum in Fig. 4a represents the system-level input-referred magnetic noise of the complete closed-loop OFG sensor. The measured noise includes the combined contributions of the magnetic core, pickup coil, excitation circuit, synchronous demodulation circuit, amplification stage, feedback loop, and data-acquisition chain. No electronic background noise from the excitation or amplification circuits was subtracted. Therefore, the reported 8 pT/√Hz at 1 Hz represents the practical system-level noise floor of the complete sensor under actual closed-loop operation.

Because all magnetic-core samples were tested using the same excitation circuit, amplification circuit, synchronous demodulation and feedback circuits, pickup coil, data-acquisition system, magnetic-shielding environment, sensitivity calibration, and equivalent magnetic-noise conversion procedure, the circuit noise serves as a common background contribution for all samples. Thus, although the circuit noise was not subtracted, the differences in low-frequency noise among different samples mainly reflect changes in core microstructure, magnetic-domain state, and dynamic magnetization loss, rather than variations in circuit parameters.

**Fig. S8** Excitation square-wave waveform measured by an oscilloscope at 125 kHz, with a time scale of 2.00 μs/div and a voltage scale of 2.00 V/div.

The sensitivity-calibration setup is shown in Fig. S9. During calibration, the OFG sensor was placed at the center of the Helmholtz coil. A series of known quasi-static axial magnetic fields were applied along the sensitive axis using a Helmholtz coil and a magnetic-field source. The output voltage after synchronous demodulation by the conditioning circuit was recorded with a digital multimeter. The magnetic-field-to-voltage sensitivity *S* was obtained from the linear fitting of the output voltage *V*_out_ versus the applied magnetic field B: *S*=d*V*_out_/ d*B.* For the optimized CoP/Ag orthogonal fluxgate, the fitted sensitivity was: *S*=153.8 kV/T. During the noise measurement, the core structure, excitation conditions, pickup coil, conditioning circuit, feedback parameters, and amplification gain were kept unchanged. Therefore, this sensitivity was used as a fixed conversion factor to convert the voltage noise spectral density into the system-level input-referred equivalent magnetic noise spectral density.

**Fig. S9** Schematic illustration of the sensitivity calibration setup.

The sensor was then placed inside a five-layer permalloy magnetic shielding cylinder. The output voltage noise spectral density, *S_V_*^1/2^(*f*), was measured using a Brüel & Kjær data acquisition system, as shown in Fig. S10. The system-level input-referred equivalent magnetic noise spectral density was calculated as: *S_B_*^1/2^(*f*)= *S_V_*^1/2^(*f*)/*S*. where *S* is the calibrated magnetic-field-to-voltage sensitivity [S14, S15]. For the optimized CoP/Ag orthogonal fluxgate, the average voltage noise density at 1 Hz was: *S_V_*^1/2^(*f*)=1.27 μV/√Hz. Thus, the corresponding system-level input-referred equivalent magnetic noise was: *S_B_*^1/2^(1 Hz)=1.27×10^−6^ V/√Hz /1.538×10^5^ V/T=8.26×10^−12^ T/√Hz≈8 pT/√Hz. Therefore, the reported noise level of 8 pT/√Hz at 1 Hz was obtained by converting the measured average voltage noise spectral density using the calibrated sensitivity.

**Fig. S10** Noise measurement setup.

**Table S11** Comparison of Sensor Parameters for Different Types of Magnetic Cores.

| Refs. | Core Type | Noise (pT/√Hz) | Core design strategy | Sensor configuration | Remarks |
| --- | --- | --- | --- | --- | --- |
| [S16] | (Co_0.94_Fe_0.06_)_72.5_Si_12.5_B_15_ | 1.5 pT/√Hz @1 Hz | Composition-optimized homogeneous CoFeSiB amorphous wire. | Orthogonal fluxgate with optimized readout. | Ultralow noise, but no composite-core design or biosensing. |
| [S17] | Co_68.2_Fe_4.3_Si_12.5_B_15_ | 50 pT/√Hz@1 Hz | Annealing-optimized glass-covered amorphous wire. | Fluxgate with signal-conditioning circuit. | Focuses on annealing-dependent noise regulation. |
| [S18] | Amorphous core | 20 pT/√Hz@10 Hz | Induced-anisotropy amorphous wire. | Magnetoimpedance magnetometer. | Mainly improves electronic readout; sensing mechanism differs from OFG. |
| [S19] | Co_68.15_Fe_4.35_Si_12.5_B_12_Cr_3_ | 300 pT/√Hz@1 Hz | Amorphous wire with induced anisotropy to improve linearity. | Open-loop parallel fluxgate. | Improved linearity, but relatively high low-frequency noise. |
| [S20] | Ni_81_Fe_19_ | 60 pT/√Hz@1 Hz | Thickness-optimized electroplated NiFe core. | Parallel fluxgate with lock-in readout. | Crystalline plated core with limited noise reduction. |
| [S21] | Fe_19_Ni_81_ | 200 pT/√Hz @1 Hz | Magnetostriction-regulated electroplated FeNi core. | Parallel fluxgate with lock-in readout. | Focuses on magnetostriction-related noise optimization. |
| [S22] | Ni_80_Fe_20_ /Cu | 59 pT/√Hz@1 Hz | Cu-core/FeNi-shell composite structure. | U-shaped composite-core OFG. | Composite design improves coupling, but crystalline FeNi limits noise reduction. |
| [S23] | [Fe_20_Ni_80_/Ti]_3_/Cu/Ti/ Fe_20_Ni_80_]_3_ | 3 nT/√Hz@1 Hz | Micro-patterned multilayer magnetic thin film | GMI microsensor. | Different sensing mechanism and higher low-frequency noise. |
| [S24] | Fe_25_Ni_75_/Fe_20_Ni_80_/Fe_15_Ni_85_ /Ag | 21pT /√Hz@1 Hz | Composition-gradient FeNi magnetic shell on Ag core. | Graded composite-core OFG. | Reduces noise by gradient-shell engineering; no biosensing demonstration. |
| [S25] | Amorphous CoZrNb film | Not reported | Microfabricated amorphous CoZrNb thin-film core. | Micro orthogonal fluxgate. | Emphasizes miniaturization; the reported sensitivity is 9.8 V/T. |
| [S26] | CoFeSiB-based amorphous core | pT-level at 1 Hz. | Homogeneous amorphous CoFeSiB core with optimized electronics. | High-sensitivity OFG. | Suitable for weak-field detection; no composite-core or biosensing route. |
| [S27] | Cu-doped CoFeSiB amorphous microwire | 45-50 pT/√Hz@1 Hz. | Cu-doped amorphous microwire with microstructure regulation. | Amorphous-microwire fluxgate. | Establishes composition–structure–noise correlation. |
| [S28] | (Co₀.₉₄Fe₀.₀₆)₇₅Si₁₅B₁₀ amorphous wire | 25 pT/√Hz @ 1 Hz; | Geometry-optimized amorphous wire core. | Parallel fluxgate. | Highlights the role of core geometry in noise control. |
| [S29] | Sc-substituted Tm₃Fe₅₋ₓScₓO₁₂ film | Not reported; | Sc-substituted garnet thin film with tuned anisotropy. | Candidate fluxgate-core material. | Material-level study; no device-level biosensing result. |
| [S30] | Co-rich amorphous microwire | 18 pT/√Hz@1 Hz | Composite-field-annealed Co-rich amorphous microwire. | Amorphous-microwire fluxgate. | Focuses on thermal-aging stability and low noise. |
| This work | Co_86_P_14_/Ag | 8 pT/√Hz@1 Hz | CoP/Ag composite core. | Closed-loop OFG with integrated conditioning circuit. | Combines low-noise composite-core design, magnetic-bead sensing, and AFP detection. |

As summarized in Table S11, fluxgate and related magnetic sensors have mainly been improved through several core-design strategies. These include optimization of homogeneous amorphous wires, electroplated NiFe/FeNi composite cores, graded magnetic shells, and microfabricated thin-film cores. Homogeneous amorphous-wire sensors, such as CoFeSiB-based OFGs, can achieve pT-level noise through alloy optimization, annealing, and signal-conditioning circuits. However, they usually do not involve composite-core phase-structure engineering or biomolecular detection. Electroplated NiFe/FeNi composite cores and graded FeNi/Ag cores can improve magnetic coupling and circumferential magnetization uniformity. Nevertheless, crystalline texture, residual stress, and domain nonuniformity may still limit their low-frequency noise performance. Miniaturized OFGs based on amorphous CoZrNb or garnet thin films are advantageous for device integration. However, their reported sensitivity or device-level noise performance remains insufficient for ultrasensitive magnetic-bead detection.

In contrast, the CoP/Ag core developed in this work features an amorphous–nanocrystalline dual-phase CoP magnetic shell on a conductive Ag substrate. This composite phase structure can reduce domain-wall pinning, promote coordinated magnetization rotation, and suppress low-frequency magnetic noise. As a result, the closed-loop OFG achieves a noise floor of 8 pT/√Hz at 1 Hz and a sensitivity of 153.8 kV/T. More importantly, this low-noise platform is further applied to magnetic-bead response measurements and AFP immunomagnetic detection. It provides a linear range of 50 fg/mL-100 ng/mL and a detection limit of 50 fg/mL. Therefore, the key feature of this work is the integration of composite-core phase-structure engineering, low-frequency noise suppression, and biomolecular detection within a single OFG platform.

***S7.1* Effect of detection-aperture size on magnetic-bead coupling**

The 3 mm detection aperture in Fig. 5c exhibits a stronger response than the 2 mm and 4 mm apertures. This indicates that the bead-detection signal is not determined solely by the bead–core distance. Instead, it is jointly governed by the local magnetic coupling strength, the effective coupling volume, and the effective number of beads contributing to signal superposition. For a single bead, the dipolar field approximately decays as 1/*r*^3^. Therefore, a smaller aperture can enhance the local coupling between near-field beads and the magnetic core. However, the 2 mm aperture has a smaller effective sampling area and coupling volume, which limits the effective bead number $N_{eff}$. As a result, its overall output is not the highest.

By contrast, the 3 mm aperture provides a better balance between strong average magnetic coupling and a sufficiently large effective coupling volume. This leads to a larger value of $N_{eff}<G(r)>$, where $<G(r)>$ denotes the average coupling factor associated with bead position and distance. Therefore, the 3 mm aperture produces the strongest magnetic-bead response. When the aperture is further increased to 4 mm, the total number of beads within the detection region increases. However, a larger fraction of beads is located farther from the magnetic core and the effective sensing region of the pickup coil. Their dipolar-field contribution decays rapidly with distance. In addition, spatial nonuniformity, local demagnetization, and shielding effects may become more pronounced, reducing the average coupling efficiency. Therefore, the aperture size is not simply better when smaller or larger. Instead, an optimal size exists, determined by the combined effects of effective coupling volume, bead number, and average magnetic coupling strength. To more clearly illustrate this trade-off, the bead–sensor coupling characteristics under different detection apertures are summarized in Table S12.

**Table S12** Comparison of magnetic-bead-sensor coupling characteristics under different detection-aperture sizes

| Aperture | Local bead-sensor coupling | Effective coupling volume | Effective bead number Neff | Overall response |
| --- | --- | --- | --- | --- |
| 2 mm | Strong | Small | Low | Limited by insufficient bead quantity and small sensing volume |
| 3 mm | Moderate-strong | Moderate | Optimal | Highest response due to balanced coupling and bead number |
| 4 mm | Weaker on average | Large | High but spatially dispersed | Reduced by distance attenuation and field nonuniformity |

***S7.2* Bias-field dependence of the magnetic-bead response**

It should be noted that the maximum output at 0.45 Oe does not mean that an individual bead has its maximum magnetic moment at this field. Rather, it indicates a favorable matching among bead magnetization, spatial distribution, and the OFG core response. Based on the Langevin magnetization relation, dipolar-field model, multi-bead structural factor, and the approximate proportionality between sensor output and bead-induced magnetic field discussed above, the bias-field-dependent response can be qualitatively expressed as: $\Delta V(H)\propto S_{OFG}(H)N_{eff}<m(H)><G(r)>F_{s}$,

where $S_{OFG}(H)$ is the effective small-signal sensitivity of the OFG, $N_{eff}$ is the effective number of coupled beads, ⟨m(H)⟩ is the average bead magnetic moment, ⟨G(r)⟩ is the geometric coupling factor, and $F_{s}$ is the structural factor reflecting bead chaining, aggregation, and shielding effects. Therefore, ΔV is not determined solely by the magnetic moment of an individual bead.

When the bias field increases from 0.45 Oe to 0.60 Oe, the magnetic moment of a single bead may continue to increase. However, stronger dipolar interactions can promote local chaining, aggregation, and shielding, thereby reducing $F_{s}$and the effective far-field superposition. Meanwhile, the differential susceptibility of the beads, dm/dH, decreases at higher bias fields, and the OFG core may deviate from its optimum small-signal operating point, leading to a lower $S_{OFG}(H)$. As a result, the net output ΔV at 0.60 Oe becomes lower than that at 0.45 Oe.

Therefore, 0.45 Oe represents the best balance among bead magnetization slope, dispersion stability, effective far-field superposition, and sensor response, rather than the field corresponding to the maximum magnetic moment of a single bead.

**Fig. S11** SEM images of 200 nm magnetic beads at different concentrations. **a** 1 ng/ml; **b** 1 μg/ml; **c** 100 μg/ml.

**Fig. S12** SEM images of 1 μm magnetic beads at different concentrations. **a** 10 ng/ml; **b** 10 μg/ml; **c** 100 μg/ml.

**Fig. S13** SEM image of magnetic beads after the immunoreaction with 100 fg/mL AFP.

**Fig. S14** SEM image of magnetic beads after the immunoreaction with 100 pg/mL AFP.

**Fig. S15** Workflow of the magnetic bead-based immunoassay for AFP detection.

***S7.3* AFP calibration fitting**

The AFP calibration curve was fitted over the concentration range of 50 fg/mL–100 ng/mL. The fitting equation is *ΔV*=4.8093log_10_(*C*_AFP_/1 fg/mL)−4.06202. with a correlation coefficient of R^2^=0.99559. Each concentration point was obtained from five repeated measurements, and the error bars represent the standard deviation (SD).

***S7.4* Additional discussion on the non-monotonic magnetic-bead response**

The non-monotonic magnetic-bead response in Figs. 5d-g can be understood as the result of competition between bead-number-induced field enhancement and the reduction in effective coupling caused by aggregation and magnetic shielding.

$$m\left( H \right)=M_{s}V_{b}L(\xi)$$

where $M_{s}$ is the effective saturation magnetization of the magnetic component in the bead, $V_{b}$ is the effective magnetic volume of the bead, and $L(\xi)=coth(\xi)-1/\xi$ is the Langevin function. The parameter $\xi$ is expressed as

$$\xi=\frac{\mu_{0}M_{s}V_{b}H}{k_{B}T}$$

where $\mu_{0}$ is the vacuum permeability, H is the applied magnetic field, kB is the Boltzmann constant, and T is the temperature. In the low-field limit ($\xi\ll1$), $L(\xi)\approx\xi/3$, and the bead moment can be approximated as

$$m\left( H \right)=\frac{\mu_{0}M_{s}^{2}V_{b}^{2}H}{{3k}_{B}T}$$

This relation indicates that the bead-induced field increases with the effective magnetic volume and the applied field.

For a single magnetized bead, the far-field magnetic flux density along the dipole axis can be approximated as

$$B_{dip}(r)\approx\frac{\mu_{0}}{4\pi}\frac{2m}{r^{3}}$$

where $r$ is the bead–sensor distance. For multiple beads within the effective sensing volume, the detectable bead-induced field can be written in a simplified form as

$$B_{eff}\approx\frac{\mu_{0}}{4\pi}\frac{2N_{eff}m}{r_{eff}^{3}}S(0)$$

where $N_{eff}$ is the effective number of magnetized beads coupled to the OFG, $r_{eff}$ is the effective bead–sensor distance, and $S(0)$ is an effective structural and coupling factor describing the influence of bead spatial correlation, aggregation, and magnetic shielding on the detectable far-field contribution. For well-dispersed beads, $S(0)$ is close to unity, and $B_{eff}$ increases mainly with $N_{eff}$. Therefore, at low concentrations, the OFG output increases with increasing bead concentration.

At higher concentrations, especially for larger beads, magnetic dipole–dipole interactions become stronger and promote spatial correlation, chain-like structures, or aggregation. The dipolar interaction energy between two magnetized beads can be expressed as

$$U_{dd}=\frac{\mu_{0}}{4\pi r_{ij}^{3}}[\boldsymbol{m}_{i}\cdot\boldsymbol{m}_{j}-3(\boldsymbol{m}_{i}\cdot\hat{r_{ij}})(\boldsymbol{m}_{j}\cdot\hat{r_{ij}})]$$

and its characteristic magnitude can be estimated as

$$U_{dd,0}=\frac{\mu_{0}m^{2}}{4\pi r_{ij}^{3}}$$

The relative importance of dipolar coupling compared with thermal disturbance can be evaluated using the dimensionless parameter

$$\Lambda=\frac{\mu_{0}m^{2}}{4\pi r_{ij}^{3}k_{B}T}$$

In the low-field regime, the bead moment can be expressed as m=αd3,

where d is the bead diameter and α is a coefficient related to the magnetic volume fraction, magnetization, and applied field. Therefore, for a fixed bead–bead distance rij, Λ can be written as

$$\Lambda=\frac{\mu_{0}\alpha^{2}d^{6}}{4\pi r_{ij}^{3}k_{B}T}$$

When the bead-bead distance scales with bead diameter, $r_{ij}=\beta d$, where $\beta$ is a spacing factor, $\Lambda$ becomes

$$\Lambda=\frac{\mu_{0}\alpha^{2}d^{3}}{4\pi\beta^{3}k_{B}T}$$

These relations indicate that the dipolar coupling strength increases rapidly with bead size, explaining why larger beads are more susceptible to spatial correlation, chain formation, and aggregation.

This interpretation is consistent with recent studies showing that magnetic iron oxide aggregates and field-induced Fe_3_O_4_ nanoparticle assemblies exhibit magnetic responses distinct from isolated particles because of interparticle dipolar interactions, structural ordering, and superstructure-induced magnetic anisotropy[S31-34].

***S7.5* Estimation of particle number density for magnetic beads with different diameters**

To further quantitatively illustrate the difference in particle number among magnetic beads with different diameters at the same mass concentration, we estimated the particle number density of beads with different diameters based on the bead mass concentrations and nominal bead diameters used in the experiments. This estimation was used to assist in understanding the concentration- and size-dependent bead response, rather than as an absolute particle-counting result.

In the calculation, the magnetic beads were approximated as spherical particles with a diameter of d. The volume of a single bead is: V_p_=πd^3^/6, The mass of a single bead is therefore: m_p_=ρV_p_=πρd^3^/6, Thus, at a bead mass concentration of *C*_MB_, the particle number density n can be expressed as: n= *C*_MB_/m_p_ = 6*C*_MB_/πρd^3^, where n is the particle number density of the magnetic beads, *C*_MB_ is the bead mass concentration, ρ is the bead density, and d is the bead diameter. In the calculation, *C*_MB_ was expressed in g/mL, d was expressed in cm, and the obtained n was expressed in particles/mL.

Because the actual magnetic component content, polymer shell fraction, and hydration state of beads may vary slightly among different manufacturers and batches, a density of ρ=1.5 g/cm^3^ was used as an approximate density for polymer-based magnetic beads in this estimation. The density value only linearly affects the absolute particle number density and does not change the relative variation trend among beads with different diameters. In other words, at the same mass concentration, the particle number density mainly follows n∝d^−3^.

The particle number densities of beads with different diameters calculated using the above method are listed in Table S13.

**Table S13** Estimated particle number density of magnetic beads with different diameters at each mass concentration

| log_10_(*C*_MB_/100 fg/mL) | *C*_MB_ | 200 nm | 300 nm | 400 nm | 500 nm | 1 μm |
| --- | --- | --- | --- | --- | --- | --- |
| 2 | 10 pg/mL | 1.59×10^3^ | 4.72×10^2^ | 1.99×10^2^ | 1.02×10^2^ | 1.27×10^1^ |
| 3 | 100 pg/mL | 1.59×10^4^ | 4.72×10^3^ | 1.99×10^3^ | 1.02×10^3^ | 1.27×10^2^ |
| 4 | 1 ng/mL | 1.59×10^5^ | 4.72×10^4^ | 1.99×10^4^ | 1.02×10^4^ | 1.27×10^3^ |
| 5 | 10 ng/mL | 1.59×10^6^ | 4.72×10^5^ | 1.99×10^5^ | 1.02×10^5^ | 1.27×10^4^ |
| 6 | 100 ng/mL | 1.59×10^7^ | 4.72×10^6^ | 1.99×10^6^ | 1.02×10^6^ | 1.27×10^5^ |
| 7 | 1 μg/mL | 1.59×10^8^ | 4.72×10^7^ | 1.99×10^7^ | 1.02×10^7^ | 1.27×10^6^ |
| 8 | 10 μg/mL | 1.59×10^9^ | 4.72×10^8^ | 1.99×10^8^ | 1.02×10^8^ | 1.27×10^7^ |
| 9 | 100 μg/mL | 1.59×10^10^ | 4.72×10^9^ | 1.99×10^9^ | 1.02×10^9^ | 1.27×10^8^ |
| 10 | 1 mg/mL | 1.59×10^11^ | 4.72×10^10^ | 1.99×10^10^ | 1.02×10^10^ | 1.27×10^9^ |

Note: A bead density of ρ=1.5 g/cm^3^ was used in the calculation. The unit is particles/mL.

As shown in Table S13, at the same mass concentration, the particle number density of magnetic beads decreases significantly with increasing bead diameter. For example, at *C*_MB_ =1 μg/mL, the estimated particle number densities of 200, 300, 400, 500 nm, and 1 μm beads are 1.59×10^8^, 4.72×10^7^, 1.99×10^7^, 1.02×10^7^, and 1.27×10^6^ particles/mL, respectively. This indicates that, at the same mass concentration, larger beads possess a higher single-particle magnetic moment but a much lower particle number density. Therefore, the OFG detection signal is not simply determined by the total bead mass, but is jointly governed by the particle number density, single-particle magnetic moment, bead dispersion state, and effective magnetic coupling between the beads and the sensor.

**Table S14** Relative particle number density, single-particle magnetic moment, and dipolar interaction index for magnetic beads with different diameters

| Bead diameter | Relative particle number density | Relative single-particle magnetic moment | Relative dipolar interaction index |
| --- | --- | --- | --- |
| 200 nm | 1.000 | 1.00 | 1.00 |
| 300 nm | 0.296 | 3.38 | 3.38 |
| 400 nm | 0.125 | 8.00 | 8.00 |
| 500 nm | 0.064 | 15.63 | 15.63 |
| 1 μm | 0.008 | 125.00 | 125.00 |

Note: All values are normalized to those of 200 nm beads. The relative particle number density was calculated as (200/d)^3^, the relative single-particle magnetic moment was calculated as (d/200)^3^, and the relative dipolar interaction index was also approximately calculated as (d/200)^3^, assuming that the bead–bead spacing scales with bead diameter.

Table S14 shows that the particle number density decreases rapidly with increasing bead diameter, whereas the single-particle magnetic moment and magnetic dipolar interaction strength increase significantly. In particular, for 500 nm and 1 μm beads, the single-particle magnetic moment and dipolar interaction index are much higher than those of 200 nm beads. Therefore, larger beads are more likely to form aggregates, chain-like structures, or local magnetic shielding at higher concentrations. This result provides quantitative scaling support for the decrease or saturation of the signal response at high bead concentrations.

**Fig. S16** The variation of output voltage with concentration under different external magnetic field intensities for magnetic beads with different diameters: (a) 300 nm;(b) 400 nm.

The additional 300 and 400 nm magnetic-bead measurements further support the size-dependent response trend. Similar to the 100, 200, 500 nm, and 1 μm beads shown in Fig. 5d-g, both 300 and 400 nm beads show a non-monotonic concentration-dependent response. The output voltage first increases with increasing bead concentration and then decreases at higher concentrations, with the maximum response appearing near 1 μg/mL. The response amplitudes of 300 and 400 nm beads are lower than that of 200 nm beads but higher than those of larger 500 nm and 1 μm beads, indicating a gradual size-dependent evolution rather than an accidental size-selection effect. These intermediate-size data bridge the original size gap between 200 and 500 nm and further support that 200 nm beads provide the most favorable response under the present sensing configuration and assay conditions.

***S7.6* Selectivity and Interference Evaluation of the AFP Immunomagnetic Assay**

In practical biosensing applications, cancer biomarkers are usually detected in complex biological matrices containing various interfering proteins. Therefore, the selectivity of the proposed AFP immunomagnetic assay was further evaluated using BSA and PSA antibody-modified magnetic beads as representative interference controls. AFP antigens were immobilized in the reaction chamber and then reacted with BSA, PSA antibody-modified magnetic beads, or AFP antibody-modified magnetic beads. The AFP antibody-modified magnetic beads corresponded to an AFP concentration of 100 ng/mL, which was the highest concentration used in the AFP concentration-dependent response measurements and was therefore selected as the positive control. The blank control produced a background response of approximately 5 mV. Thus, the output voltage of each sample was corrected by subtracting the blank-control voltage, and the resulting value was defined as *ΔV*. The BSA sample induced only a negligible response of 3.40±0.70 mV, while the PSA antibody-modified magnetic beads generated a response of 8.20±0.82 mV, which was much lower than that obtained from the AFP antibody-modified magnetic beads, 34±1.21 mV, as shown in Fig. S17. These results indicate that non-target protein interference and nonspecific magnetic-bead binding have limited effects on the AFP detection signal, supporting the preliminary selectivity of the proposed immunomagnetic sensing method. All measurements were repeated at least five times, and the data are presented as mean values with standard deviations.

**Fig. S17** Interference analysis of the AFP immunomagnetic assay.

***S7.7* Effect of magnetic-bead aggregation on the concentration-dependent OFG response**

Magnetic-bead aggregation can affect the OFG response even without obvious bead rotation or translational motion during measurement [S32, S35]. When the beads are well dispersed, the bead-induced stray fields can more effectively contribute to the far-field signal detected by the OFG. Once the beads form aggregates, their magnetic dipoles become spatially correlated, and internal demagnetizing fields and local magnetic shielding are enhanced. Therefore, the detectable far-field contribution is no longer proportional to the total bead number, leading to weakened effective bead–sensor coupling at high concentrations [S31, S36]. This explains the signal saturation or decrease after the maximum response. Accordingly, the four concentration regimes are used to describe the continuous evolution of the bead response from sparse-bead response, bead-number-dominated signal enhancement, and peak response to aggregation- and shielding-dominated signal attenuation.

**Table S15** The performance of different magnetic biosensors in detecting alpha-fetoprotein

| Analysis method | Linear range | Detection limit |
| --- | --- | --- |
| GMI Sensor[S37] | 100 fg/mL-1μg/mL | 100 fg/mL |
| GMI Sensor [S38] | / | 0.2 ng/ml |
| Fluxgate sensor[S39] | / | 1 pg/ml |
| GMR Sensor [S40] | / | 20 ng/mL |
| GMR Sensor [S41] | 1 ng/mL-1000 ng/mL | 0.52 ng/mL |
| This Work | 50 fg/mL-100 ng/mL | 50 fg/mL |

**Supplementary References**

[S1] A. Vansteenkiste, J. Leliaert, M. Dvornik, M. Helsen, F. Garcia-Sanchez et al., The design and verification of MuMax3. AIP Adv. **4**(10), 107133 (2014). <https://doi.org/10.1063/1.4899186>
[S2] A. Vansteenkiste, B. Van de Wiele, MuMax: a new high-performance micromagnetic simulation tool. J. Magn. Magn. Mater. **323**(21), 2585–2591 (2011). <https://doi.org/10.1016/j.jmmm.2011.05.037>
[S3] N. Eliaz, G. Weisman, A. Kohn, G. Levi, B.A. Rosen et al., Electrodeposition of amorphous cobalt–phosphorus coating. Materials **18**(21), 4883 (2025). <https://doi.org/10.3390/ma18214883>
[S4] S. Bance, J. Fischbacher, T. Schrefl, I. Zins, G. Rieger et al., Micromagnetics of shape anisotropy based permanent magnets. J. Magn. Magn. Mater. **363**, 121–124 (2014). <https://doi.org/10.1016/j.jmmm.2014.03.070>
[S5] A. Ducevic, F. Bruckner, C. Abert, C. Vogler, D. Suess, Micromagnetic simulations with periodic strayfield calculation of soft magnetic composite-materials. Sci. Rep. **15**(1), 17782 (2025). <https://doi.org/10.1038/s41598-025-01881-8>
[S6] J.-Q. Wang, L.J. Song, J.T. Huo, M. Gao, Y. Zhang, Designing advanced amorphous/nanocrystalline alloys by controlling the energy state. Adv. Mater. **36**(37), e2311406 (2024). <https://doi.org/10.1002/adma.202311406>
[S7] R. Stolz, M. Schiffler, M. Becken, A. Thiede, M. Schneider et al., SQUIDs for magnetic and electromagnetic methods in mineral exploration. Miner. Econ. **35**(3–4), 467–494 (2022). <https://doi.org/10.1007/s13563-022-00333-3>
[S8] Y. Yan, J. Lu, S. Zhang, F. Lu, K. Yin et al., Three-axis closed-loop optically pumped magnetometer operated in the SERF regime. Opt. Express **30**(11), 18300–18309 (2022). <https://doi.org/10.1364/OE.458367>
[S9] A. Kanno, N. Nakasato, M. Oogane, K. Fujiwara, T. Nakano et al., Scalp attached tangential magnetoencephalography using tunnel magneto-resistive sensors. Sci. Rep. **12**(1), 6106 (2022). <https://doi.org/10.1038/s41598-022-10155-6>
[S10] B. Spetzler, C. Bald, P. Durdaut, J. Reermann, C. Kirchhof et al., Exchange biased delta-E effect enables the detection of low frequency pT magnetic fields with simultaneous localization. Sci. Rep. **11**, 5269 (2021). <https://doi.org/10.1038/s41598-021-84415-2>
[S11] K. Greene, C. Hansen, B.B. Narod, R. Dvorsky, D.M. Miles, Tesseract–a high-stability, low-noise fluxgate sensor designed for constellation applications. Geosci. Instrum. Method. Data Syst. **11**(2), 307–321 (2022). <https://doi.org/10.5194/gi-11-307-2022>
[S12] P. Bera, H. Seenivasan, K.S. Rajam, V.K. William Grips, Characterization of amorphous Co–P alloy coatings electrodeposited with pulse current using gluconate bath. Appl. Surf. Sci. **258**(24), 9544–9553 (2012). <https://doi.org/10.1016/j.apsusc.2012.05.115>
[S13] B. Sarac, Y.P. Ivanov, A. Chuvilin, T. Schöberl, M. Stoica et al., Origin of large plasticity and multiscale effects in iron-based metallic glasses. Nat. Commun. **9**, 1333 (2018). <https://doi.org/10.1038/s41467-018-03744-5>
[S14] M. Butta, I. Sasada, Sources of noise in a magnetometer based on orthogonal fluxgate operated in fundamental mode. IEEE Trans. Magn. **48**(4), 1508–1511 (2012). <https://doi.org/10.1109/TMAG.2011.2173177>
[S15] H. Kang, H. Jin, Y. Jang, S. Lee, H. Park et al., Performance test of search coil sensors with different CoreTypes. J. Astron. Space Sci. **41**(3), 139–148 (2024). <https://doi.org/10.5140/jass.2024.41.3.139>
[S16] M. Butta, M. Janošek, J. Pařez, A.V. Inchausti, H. Chiriac, An improved composition of CoFeSiB alloy for orthogonal fluxgates. Sensors **22**(6), 2162 (2022). <https://doi.org/10.3390/s22062162>
[S17] B. Yan, W. Zhu, Y. Sun, Z. Lu, X. Zhuang et al., Discussion on the relationship between sensor noise and glass-covered amorphous magnetic wires annealing temperature. IEEE Sens. J. **24**(7), 9792–9799 (2024). <https://doi.org/10.1109/JSEN.2024.3363619>
[S18] I. Akita, T. Kawano, H. Aoyama, S. Tatematsu, M. Hioki, An automatic loop gain enhancement technique in magnetoimpedance-based magnetometer. IEEE J. Solid State Circuits **57**(12), 3704–3715 (2022). <https://doi.org/10.1109/JSSC.2022.3202224>
[S19] C. Mu, L. Pan, H. Ma, M. Su, A. He et al., Enhancing linearity of open-loop parallel fluxgate sensors *via* induced anisotropy in amorphous wires. J. Alloys Compd. **1027**, 180636 (2025). <https://doi.org/10.1016/j.jallcom.2025.180636>
[S20] M. Butta, Effect of thickness of electroplated NiFe cores on the noise of fluxgates. Acta Phys. Pol. A **131**(4), 756–758 (2017). <https://doi.org/10.12693/aphyspola.131.756>
[S21] M. Butta, M. Janosek, P. Ripka, L. Kraus, R. El Kammouni, Influence of magnetostriction of NiFe electroplated film on the noise of fluxgate. IEEE Trans. Magn. **50**(11), 4006504 (2014). <https://doi.org/10.1109/TMAG.2014.2327105>
[S22] X. Pu, Q. Zhou, G. Chai, D. Gao, Optimization of U-shaped Cu@ Fe_20_Ni80 core for orthogonal fluxgate sensor. J. Magn. Magn. Mater. **592**, 171822 (2024). <https://doi.org/10.1016/j.jmmm.2024.171822>
[S23] E. Fernández, A. García-Arribas, J.M. Barandiarán, A.V. Svalov, G.V. Kurlyandskaya et al., Equivalent magnetic noise of micro-patterned multilayer thin films based GMI microsensor. IEEE Sens. J. **15**(11), 6707–6714 (2015). <https://doi.org/10.1109/JSEN.2015.2463254>
[S24] X. Pu, Z. Yu, J. Gao, M. Zhong, L. Wang et al., Heterogeneous assembly of graded magnetic composite wires for noise suppression in orthogonal fluxgate. Sens. Actuat. A Phys. **394**, 116899 (2025). <https://doi.org/10.1016/j.sna.2025.116899>
[S25] K.-W. Kim, S.-M. Hong, D. Lee, K.-H. Shin, S.H. Lim, Micro orthogonal fluxgate sensor fabricated with amorphous CoZrNb film. Sensors **25**(16), 5022 (2025). <https://doi.org/10.3390/s25165022>
[S26] P. Priftis, S. Angelopoulos, A. Ktena, E. Hristoforou, Development of a high-sensitivity orthogonal fluxgate sensor. J. Magn. Magn. Mater. **590**, 171646 (2024). <https://doi.org/10.1016/j.jmmm.2023.171646>
[S27] B. Wang, W. Xu, X. Zheng, S. Jiang, Z. Yi et al., Performance of fluxgate magnetometer with Cu-doped CoFeSiB amorphous microwire core. Sensors **24**(1), 309 (2024). <https://doi.org/10.3390/s24010309>
[S28] D. Hrakova, P. Ripka, M. Butta, Sensitivity and noise of parallel fluxgate sensor with amorphous wire cores. J. Magn. Magn. Mater. **563**, 169981 (2022). <https://doi.org/10.1016/j.jmmm.2022.169981>
[S29] S. Yang, D. Zhang, Y. Zhang, H. Li, Z. Li et al., In-plane magnetic properties and anisotropy of scandium substituted thulium iron garnet thin films for fluxgate magnetometer. J. Magn. Magn. Mater. **598**, 171993 (2024). <https://doi.org/10.1016/j.jmmm.2024.171993>
[S30] L. Pan, Z. Cui, L. Pan, X. Jia, M. Su et al., Composite-field annealing enables superior high-temperature noise stability in fluxgate sensors with co-rich amorphous microwires. Rare Met. **45**(2), e70093 (2026). <https://doi.org/10.1002/rar2.70093>
[S31] M. Nedylakova, J. Medinger, G. Mirabello, M. Lattuada, Iron oxide magnetic aggregates: Aspects of synthesis, computational approaches and applications. Adv. Colloid Interface Sci. **323**, 103056 (2024). <https://doi.org/10.1016/j.cis.2023.103056>
[S32] A. Basu, M.R. Clary, J.B. Tracy, C.K. Hall, O.D. Velev, Spatially confined assembly and immobilization of hierarchical nanoparticle architectures inside microdroplets in magnetic fields. ACS Nano **18**(30), 19814–19827 (2024). <https://doi.org/10.1021/acsnano.4c06285>
[S33] J. Mohapatra, P. Joshi, H. Abbas, M. Gusenbauer, K. Bian et al., Superstructure magnetic anisotropy in Fe(3)O(4) nanoparticle chains. Nat. Commun. **16**(1), 5723 (2025). <https://doi.org/10.1038/s41467-025-60888-x>
[S34] G.M. Nabar, A.V. Dehankar, E. Jergens, B.B. Hansen, E. Johnston-Halperin et al., Structural interactions in polymer-stabilized magnetic nanocomposites. Soft Matter **20**(18), 3732–3741 (2024). <https://doi.org/10.1039/d4sm00008k>
[S35] A. Sayad, E. Skafidas, P. Kwan, Magneto-impedance biosensor sensitivity: effect and enhancement. Sensors **20**(18), 5213 (2020). <https://doi.org/10.3390/s20185213>
[S36] T.B.G. Hansen, C.D. Damsgaard, B.T. Dalslet, M.F. Hansen, Theoretical study of in-plane response of magnetic field sensor to magnetic beads magnetized by the sensor self-field. J. Appl. Phys. **107**(12), 124511 (2010). <https://doi.org/10.1063/1.3366717>
[S37] Y. Zhu, Q. Zhang, X. Li, H. Pan, J. Wang et al., Detection of AFP with an ultra-sensitive giant magnetoimpedance biosensor. Sens. Actuat. B Chem. **293**, 53–58 (2019). <https://doi.org/10.1016/j.snb.2019.05.004>
[S38] L. Guo, S. Zhi, X. Sun, C. Lei, Y. Zhou, Ultrasensitive detection of bioanalytes based on signal amplification of coil-integrated giant magnetoimpedance biosystems. Sens. Actuat. B Chem. **247**, 1–10 (2017). <https://doi.org/10.1016/j.snb.2017.01.095>
[S39] Z. Yang, J. Lei, X.-C. Sun, C. Lei, Y. Zhou et al., A Dynabeads-labeled immunoassay based on a fluxgate biosensor for the detection of biomarkers. Anal. Methods **7**(6), 2391–2398 (2015). <https://doi.org/10.1039/c4ay02727b>
[S40] C. Yao, E. Ng, S.X. Wang, An automated and mobile magnetoresistive biosensor system for early hepatocellular carcinoma diagnosis. Biosens. Bioelectron. **202**, 113982 (2022). <https://doi.org/10.1016/j.bios.2022.113982>
[S41] Y. Gao, W. Huo, L. Zhang, J. Lian, W. Tao et al., Multiplex measurement of twelve tumor markers using a GMR multi-biomarker immunoassay biosensor. Biosens. Bioelectron. **123**, 204–210 (2019). <https://doi.org/10.1016/j.bios.2018.08.060>
